# Cortical region–specific sleep homeostasis in mice: effects of time of day and waking experience

**DOI:** 10.1093/sleep/zsy079

**Published:** 2018-04-25

**Authors:** Mathilde C C Guillaumin, Laura E McKillop, Nanyi Cui, Simon P Fisher, Russell G Foster, Maarten de Vos, Stuart N Peirson, Peter Achermann, Vladyslav V Vyazovskiy

**Affiliations:** 1 Nuffield Department of Clinical Neurosciences, University of Oxford, Oxford, United Kingdom; 2 Department of Physiology, Anatomy and Genetics, University of Oxford, Oxford, United Kingdom; 3 Department of Engineering Science, University of Oxford, Headington, United Kingdom; 4 Institute of Pharmacology and Toxicology, University of Zurich, Zurich, Switzerland

**Keywords:** sleep homeostasis, wakefulness, behavior, sleep deprivation

## Abstract

Sleep–wake history, wake behaviors, lighting conditions, and circadian time influence sleep, but neither their relative contribution nor the underlying mechanisms are fully understood. The dynamics of electroencephalogram (EEG) slow-wave activity (SWA) during sleep can be described using the two-process model, whereby the parameters of homeostatic Process S are estimated using empirical EEG SWA (0.5–4 Hz) in nonrapid eye movement sleep (NREMS), and the 24 hr distribution of vigilance states. We hypothesized that the influence of extrinsic factors on sleep homeostasis, such as the time of day or wake behavior, would manifest in systematic deviations between empirical SWA and model predictions. To test this hypothesis, we performed parameter estimation and tested model predictions using NREMS SWA derived from continuous EEG recordings from the frontal and occipital cortex in mice. The animals showed prolonged wake periods, followed by consolidated sleep, both during the dark and light phases, and wakefulness primarily consisted of voluntary wheel running, learning a new motor skill or novel object exploration. Simulated SWA matched empirical levels well across conditions, and neither waking experience nor time of day had a significant influence on the fit between data and simulation. However, we consistently observed that Process S declined during sleep significantly faster in the frontal than in the occipital area of the neocortex. The striking resilience of the model to specific wake behaviors, lighting conditions, and time of day suggests that intrinsic factors underpinning the dynamics of Process S are robust to extrinsic influences, despite their major role in shaping the overall amount and distribution of vigilance states across 24 hr.

**Statement of Significance**

The notion that sleep–wake history determines the levels of homeostatic sleep pressure, referred to as Process S, has been widely used to obtain insights into sleep regulatory mechanisms. Although time awake is considered the main variable affecting sleep need, investigating the role of additional extrinsic influences on the dynamics of Process S remains essential to understand its neurophysiological substrates. We used a combination of experimental and modeling approaches to investigate the influence of waking behavior and time of day on sleep homeostasis. Unexpectedly, the performance of our model was robust across experimental conditions, suggesting that the mechanisms underlying Process S dynamics are resilient to external factors, which are mostly responsible for regulating the amount and daily distribution of waking and sleep.

## Introduction

The propensity for wake and sleep across the 24 hr day is thought to be determined by the time of day, lighting conditions, specific waking activities, preceding sleep–wake history, and homeostatically regulated needs such as hunger [1–7]. During nonrapid eye movement sleep (NREMS), slow waves (0.5–4 Hz) dominate the electroencephalogram (EEG) over the cortical surface [8, 9]. Slow waves during sleep are considered a marker of sleep “intensity,” and in both animals and humans it has been shown that the levels of slow-wave activity (SWA, EEG power in the slow-wave frequency range) change as a function of prior waking and sleep. Specifically, SWA displays a global declining trend during sleep, and the level of EEG SWA at the onset of the first NREMS episode depends on prior waking duration [10–12], reflecting the homeostatic regulation of sleep. There are numerous known mechanisms responsible for the daily distribution of waking and sleep and the homeostatic dynamics of SWA; yet, a fundamental and unanswered question is whether these two aspects of sleep regulation are independent.

Homeostatic regulation involves a sensor and an effector, which monitor changes in regulated variables and trigger adequate responses to keep them within a given range [13]. How these relate to homeostatic regulation of sleep intensity is unknown, but evidence suggests that sleep is implicated in a variety of restorative processes related to energy homeostasis, synaptic homeostasis, and prophylactic cellular maintenance [14–19]. At the cellular and molecular levels, it has been shown that the levels of adenosine increase progressively in the basal forebrain and in some cortical areas during wake [14, 20, 21], which may influence activity in subcortical circuits responsible for sleep control [16], or directly inhibit specific neuronal populations in the cortex [14, 16]. Furthermore, it has been proposed that specific waking activities occurring in local cortical areas may contribute to the local accumulation of sleep need [16, 22, 23]. Consistent with this, several molecular markers of synaptic strength or neuronal excitability change across waking and sleep [24–27], although it is unclear whether they are directly regulated by states of vigilance, or merely represent an epiphenomenon of other processes not implicated directly in sleep regulation [28–31].

Although much progress has been made in understanding molecular, cellular, and network mechanisms underlying sleep regulation [32–40], its temporal dynamics remain poorly understood, and computer modeling approaches may provide important insights. The two-process model has been widely used as a framework to address the contribution of the homeostatic component (Process S) and the circadian component (Process C) to the regulation of sleep [2, 41]. In humans, Process C reflects the daily variations of sleep propensity that keeps track of environmental time. This is controlled by the circadian pacemaker located in the suprachiasmatic nuclei of the hypothalamus [42]. Process S tracks instantaneous sleep need, and empirical EEG SWA—specifically in NREMS—is traditionally used to estimate the parameters necessary to simulate Process S.

In its original formulation, the two-process model assumed the presence of a single circadian pacemaker, which constrained the variation of Process S between two thresholds that were subject to a circadian oscillation [2, 41]. A later, so called “elaborated,” version of the model accounted for the intraepisodic dynamics of SWA in humans and assumed that the levels of SWA determine the changes in Process S [43, 44]. The applicability of the original two-process model to rodent data was tested and shown as early as 1991 [45], and in 2000, the model was first used in mice, to show how the amount and distribution of sleep episodes differ between strains [46]. However, to date, the elaborated version of the model has not been used in rodents.

Although originally the model tackled sleep homeostasis as a global process, it soon became apparent that SWA shows pronounced topographical variations, suggesting that cortical regions differ in their dynamics [47–51]. Several studies in rodents have shown a frontal predominance of SWA during sleep [52–54]. Although the underlying mechanisms are not fully understood, evidence suggests that preceding behavior, as well as anatomical differences between cortical regions, may be important contributing factors [22, 26, 55, 56]. It was therefore proposed that the dynamics of Process S should also differ between cortical regions [50], which has since been supported by several recent studies [47, 51]. Furthermore, sleep–wake distribution and the levels of SWA are markedly affected by lighting conditions as well as other factors [3, 57, 58], and it has been suggested that SWA may be influenced by a varying drive for wakefulness, which changes with circadian phase [59].

Several animal studies have used a simplified version of the model to describe the time course of Process S on a time scale of hours [45, 46, 58, 59]. However, SWA shows distinct region-specific dynamics on a time scale of minutes, where both long-term history and immediate preceding state play a role [60]. These important aspects have not been previously tackled using a modeling approach in laboratory rodents. Starting from the 1993 model published by Achermann et al. [44] (which used human data), we adapted the parameters and equations to fit baseline EEG recordings performed in mice from two different cortical regions. We then addressed the performance of the model in predicting SWA levels in several experimental conditions, including learning a novel motor skill or sleep deprivation achieved by providing novel objects. The model performed well across conditions, and neither waking experience nor time of day was found to have a significant influence on the fit between data and simulation. Our results suggest that the intrinsic mechanisms underlying the dynamics of Process S are robust to extrinsic influences, and that those extrinsic factors instead determine the timing and duration of consolidated periods of waking and sleep.

## Materials and Methods

### Animals

Adult male C57BL/6J mice (Harlan UK Ltd., Bicester, United Kingdom) were used in this study, with data obtained under three experimental conditions:

- *Regular-wheel (RW*): The first group (*n* = 7, age = 13 ± 2 weeks, mean ± SD) was used to investigate the temporal dynamics and regional differences in Process S during 48 hr of undisturbed baseline conditions (12:12 hr light/dark cycle) with access to a standard running wheel.
- *Complex*-*wheel (CW*): In the second group (*n* = 7, age = 15 ± 1 weeks, mean ± SD), complex wheels were used (i.e. wheels missing rungs with an irregular pattern, Supplementary Figure S1a) to investigate the influence of waking behavior, such as learning a novel motor skill, on the performance of the model. In this experiment, following an undisturbed 24 hr recording when a RW was available (which the animals had been habituated to for at least 1 week), a CW was introduced and recordings were continued for at least 24 hr. One animal did not run at all and showed no sustained waking in the dark phase when the CW was introduced and was therefore excluded from some of the analyses wherever appropriate. No significant difference in time spent running was observed between RW and CW nights (ANOVA test, factor “day”; entire dark phase: *F*(1,6) = 1.006, *p* = 0.355; long wake bout only: *F*(1,6) = 0.199, *p* = 0.671).
- *Exploratory wakefulness (EW):* In the third group of animals (*n* = 7, age = 17 ± 5 weeks, mean ± SD), an undisturbed 24 hr recording was followed by 6 hr sleep deprivation (from light onset) and 18 hr recovery recordings. This group was used to determine whether predictions of the model are affected by the time of day and the nature of waking behavior.

The number of animals used in this study was in line with previous studies where computer simulations or regional differences in SWA were investigated [45, 46, 52, 56, 58, 59]. No power calculations were employed here as the elaborated version of the model has not been used previously in rodents, and the effect sizes were uncertain.

Age was similar between groups, as revealed by a one-way ANOVA (*F*(2,18) = 2.57, *p* = 0.104). Other general experimental conditions were as described previously [60–62]. Briefly, animals were individually housed in custom-made clear Plexiglas cages (dimensions: 20.3 × 32 × 35 cm^3^) placed in ventilated, sound-attenuated Faraday chambers (Campden Instruments, Loughborough, UK). Mice had free access to running wheels (Campden Instruments, Loughborough, UK, wheel diameter: 14 cm), or to CWs on the specified days. Recordings started a minimum of 4 days after the mice were cabled for habituation to recording cables and running wheels. Mice were kept at 22 ± 1°C with a humidity level of 60 ± 10% and maintained on a 12:12 hr light-dark cycle (light levels ∼ 120–180 lux). Food and water were available *ad libitum*. All work was carried out under a UK Home Office PPL in accordance with Animal [Scientific Procedures] Act 1986 and the University of Oxford’s guidelines.

### Surgical procedure

The surgical procedure was performed using aseptic techniques and under isoflurane anesthesia (3%–5% for induction, 1%–2% for maintenance). Animals were head-fixed using a stereotaxic frame (David Kopf Instruments, CA, USA). Prior to surgery, mice were administered with Metacam (1–2 mg/kg, s.c.) and dexamethasone (0.2 mg/kg, s.c.). The surgery consisted of the implantation of EEG and electromyography (EMG) electrodes, which has been described previously [60, 62]. As reported in Fisher et al. [62], EEG screws were placed in the frontal (motor area, anteroposterior +2 mm, mediolateral 2 mm) and occipital (visual area, V1, anteroposterior −3.5 to −4 mm, mediolateral 2.5 mm) cortical regions, a reference screw was placed above the cerebellum and an additional screw was placed in the opposite occipital bone to ensure stability of the implant. Finally, two stainless-steel wires were implanted on each side of the nuchal muscle to record the EMG. Some of the animals were also implanted with a microwire array in the frontal cortex to record neuronal activity, which is not analyzed here. Postoperatively, animals were administered with saline (0.1 mL/20 g, s.c.) to compensate for fluid loss and were provided with thermal support. Metacam (1–2 mg/kg) and dexamethasone (0.2 mg/kg) were administered orally for a period of at least 3 days and 2 days following surgery, respectively. The animals were left undisturbed for at least 2 weeks before being cabled.

### Data acquisition and signal processing

Data acquisition was performed using the Multichannel Neurophysiology Recording System (Tucker-Davis Technologies Inc. [TDT], USA). EEG and EMG were continuously recorded, filtered between 0.1 and 100 Hz, amplified (PZ5 NeuroDigitizer pre-amplifier, TDT), and stored on a local computer at a sampling rate of 256.9 Hz, before being resampled offline at 256 Hz. Using custom-written Matlab (The MathWorks Inc., USA) scripts, signals were converted to .txt format. Txt files were then transformed to European Data Format (EDF) using the open-source Neurotraces software. For further details on signal processing, see Vyazovskiy et al. [63], Cui et al. [60], and Fisher et al. [62].

### Scoring of vigilance states

Vigilance states were scored by visual inspection of consecutive 4 s epochs (4 s epoch = 1 ts, ts standing for “time step”), using *SleepSign* software (Kissei Comtec Co., Nagano, Japan). To facilitate scoring, two EEG channels (frontal and occipital) and EMG were displayed. For each animal, at least four consecutive 12 hr light and dark periods were scored, starting with a light period. Vigilance states were defined as waking (low-amplitude high-frequency EEG with a high level of EMG activity), NREMS (presence of slow-waves, high-amplitude, and low-frequency EEG with a low level of EMG activity), rapid eye movement sleep (REMS, low-amplitude, high-frequency EEG with a low level of EMG activity), or brief awakenings (periods of no more than five consecutive 4 s epochs of waking, occurring during NREMS or immediately following REMS episodes, and characterized by the presence of EMG activity). If any of the EEG channels were contaminated by artifacts, the epoch was scored as artifactual (i.e. the vigilance state was defined, but SWA values were ignored in subsequent analyses). In the RW/CW groups, artifactual epochs represented 2.8 ± 0.6% (mean ± SEM) of all epochs. The percentages of artifactual epochs within each vigilance state was below 6% (Wake: 4.4 ± 1.2%; NREMS: 1.5 ± 1.2%; REMS: 1.3 ± 1.0%, mean ± SEM). Once the scoring was complete, EEG power density spectra were computed for all 4 s epochs by a fast Fourier transform (Hanning window; 0.25 Hz resolution; frequency range 0 to 20 Hz) and exported from SleepSign for further analysis.

### Calculation of EEG slow-wave activity

SWA was defined as EEG power in the slow-wave range (0.5–4 Hz). Unless otherwise stated, values were subsequently normalized to the mean over all NREMS epochs during the baseline recording (baseline 48 hr for the RW and CW conditions, first 24 hr only for the EW condition, as described below).

### RW recordings

This cohort of animals was left undisturbed with access to a standard running wheel. Therefore, in this group, the “baseline” recordings that we will refer to correspond to 48 hr that will be used for the modeling (see below).

### CW recordings

In seven animals, 24 hr baseline recordings were followed by a 24 hr recording during which the animals had access to a “complex wheel,” i.e. a wheel with rungs missing in an irregular pattern (Supplementary Figure S1a). In this cohort, as baseline recordings had been performed for at least 48 hr before the replacement of the standard wheel with a CW, parameters were optimized (see below) using the 2 days prior to the RW being exchanged for the CW. For all subsequent analyses, the dataset consisted of the last day of access to a RW (i.e. prior to the wheel being exchanged) and of the first day the animals had access to a CW.

### EW recordings

In the group of animals that underwent sleep deprivation, after at least 24 hr of baseline recordings, a 6 hr sleep deprivation was performed starting at light onset. As the mice were kept awake during the light phase, which is their habitual quiet phase, this provided an opportunity to investigate the impact of the time of day on the dynamics of Process S and on the performance of the model. The sleep deprivation was performed starting from light onset, as our aim was to test the ability of our model to accurately predict the increase in SWA levels which follows a long period of waking during the time of day when the animals are typically asleep. Given that long periods of waking naturally occur in the dark, performing the sleep deprivation at dark onset would not have provided us with a condition significantly deviating from baseline condition, against which the parameters have been optimized. Animals were kept awake by giving them new objects to explore or, if the animals were getting very drowsy, by tapping gently on the cage. The procedure was successful as only 1.6 ± 0.2% (mean ± SEM) of time was spent asleep (NREMS and REMS episodes included) during the 6 hr of EW. Although mice became gradually more and more drowsy, they still engaged with object exploration until the end of the 6 hr procedure of sleep deprivation. Importantly, our aim was not to merely sleep deprive the mice, but to make them engage in exploration, to contrast this behavior against wheel-running (RW and CW cohorts). Following sleep deprivation, animals were left undisturbed. The 48 hr EW datasets we will refer to here consist of 24 hr of baseline followed immediately by 6 hr of EW and 18 hr of recovery. However, as the optimization process required 48 hr of baseline (BL) recordings (see below), we duplicated the first 24 hr to estimate the parameters in this cohort, but all subsequent analyses were done on the dataset consisting of 24 hr baseline followed by the sleep deprivation and recovery day. We chose to duplicate the 24 hr of baseline in the EW group to keep an approach similar to the RW and CW groups where 48 hr of baseline were available. The reasons to optimize against 48 hr instead of 24 hr in all three cohorts were that previous work [44, 64] and our initial simulations showed that this yields a better fit and a more stable and accurate estimation of parameter values.

### Selection of NREMS episodes

In most analyses (including the optimization process), we refer to “NREMS episodes” [65] as periods of NREMS longer than 1 min, allowing for short interruptions of no more than 4 ts (=16 s) at a time. These are to be differentiated from “sleep bouts” describing periods of sustained sleep, including both NREMS and REMS and brief awakenings. The choice of keeping NREMS episodes of at least 1 min was based on previous findings [66, 67] and our observation that very short NREMS episodes of up to 1 min duration have lower levels of SWA on average, not reflecting the level of sleep pressure, as the shorter episode length does not allow a full build-up of SWA. To analyze the intraepisodic dynamics of SWA during NREMS episodes, it was sometimes necessary to differentiate between shorter and longer NREMS bouts. In this paper, we refer to episodes that were at least 3 min in duration as long NREMS episodes.

### Description of the model

The model was based on the following equations, which were adapted from Achermann et al. [44]:

(1) **Slow-wave activity (SWA(t)):** Empirical SWA (and therefore simulated SWA) is always expressed—unless otherwise stated—as a percentage of mean SWA across NREMS in the baseline period used for optimizations (RW and CW: 48 hr, EW: 2 × 24 hr—as described above),

$$\;\frac{\text{dSWA}}{\text{dt}}\;=\;rc.\;SWA\;.\;\;\frac{\text{S}}{\text{SU}}\;.\;\left(1-\;\frac{\text{SWA}}{\text{S}}\;\right).\;\left(1-\mathrm{WT}\left(t\right)\right).\;(1-\mathrm{REMT}(t))$$

$$–\;fc_{R}.\;\left(SWA\;–\;SWA_{L}\right).\;REMT(t)$$

$$–\;fc_{W}.\;\left(SWA\;–\;SWA_{L}\right).\;WT(t)$$

(2) REMS (REMT(t)):

$$REMT\left(t\right)=\{\begin{array}{c} 1\;during\;REMS\;'trigger'\;(see\;below)\\ 0\;otherwise\; \end{array}$$

(3) Waking (WT(t)):

$$WT\left(t\right)=\{\begin{array}{c} 1\;during\;wake\;'trigger^{'}(see\;below)\\ 0\;otherwise \end{array}$$

(4) Process S (S(t)):

$$\frac{\text{dS}}{\text{dt}}\;=\;-\;gc.\;SWA\;+\;\left(S_{U}\;–\;S\right).\;rs$$

Definitions and further descriptions of the parameters can be found in Table 1. The time step used here is 4 s, whereas the original 1993 model [44] used 1 min time steps. This change was necessary to account for the more frequent and rapid alternation between vigilance states observed in mice in comparison with human subjects. The differential equation (1) governs the time course of SWA and shows a sigmoid-like build-up component followed by two fall components depending on the REMS (REMT) and wake (WT) triggers. Compared with REMS, a REMT episode starts t_a_ epochs before a REMS episode and lasts t_p_ epochs longer (Supplementary Figure S2). This works similarly for WT compared with wake (t_a_ being replaced by t_aw_ and t_p_ by t_pw_). Allowing the model to anticipate the occurrence of REMS and wake by introducing the trigger functions REMT and WT, which are directly derived from the data (Supplementary Figure S2), enables us to obtain a better fit, just as it did in the original version of the model from Achermann et al. [44]. Equation (4) governing the time course of Process S displays a decline component proportional to the level of SWA and a permanently activated rise component. The terms in grey shade in equation (1) have been added to the original equations [44] to allow for a steeper fall of SWA levels at transitions from NREMS to REMS or to wake, as is observed in mice, as well as to correct for a deviation in the saturating increase of Process S during wake. Without those additional terms, the decline of SWA at the end of a NREMS episode was initially steep and then much attenuated as SWA levels became lower; this did not reflect the variations we observed in the empirical data. “Switching off” the build-up component of equation (1) as soon as an animal enters REMS or wake—as those grey-tone multiplication factors allow—provided a better fit between data and simulation. The differential equations were implemented in Matlab; as our system of equations was stiff (mainly due to state transitions), we used the “ode15s” solver. The solver was constrained to take maximal steps of 4 s (to ensure that at least one data point was obtained for each 4 s epoch), but the solver was then left free to take smaller steps if necessary. Once the final 48 hr solution from ode15s was obtained, data points belonging to the same 4 s epoch were averaged to obtain a single value per 4 s epoch. Annex functions were written to calculate REMS trigger (REMT) and wake trigger (WT) (Table 1).

**Table 1.** Summary of the parameters and functions used in the model

Parameters	Definition	Description	Value used for the simulations*
rc	Rise constant	Determines the rise of SWA within NREMS episodes	0.5
fc_R_	Fall constant REMT	Determines the fall of SWA triggered by REMT	(0.1; 0.2; 0.3)
fc_W_	Fall constant WT	Determines the fall of SWA triggered by WT	0.2
SWA_L_	Lower asymptote of SWA		20
SWA_0_	Initial level of SWA at the beginning of the simulation		Adjusted for each animal and derivation
REMT(t)	REMS trigger	Activates the fall of SWA	1 or 0 (see equations)
t_a_	Advance of REMT	Advance of REMT with respect to the onset of empirical REMS	8
t_p_	Prolongation of REMT	Prolongation of REMT with respect to the duration of empirical REMS	6
WT(t)	Wake trigger	Activates the fall of SWA	1 or 0 (see equations)
t_aw_	Advance of WT	Advance of WT with respect to the onset of empirical waking	8
t_pw_	Prolongation of WT	Prolongation of WT with respect to the duration of empirical waking	4
gc	Gain constant	Determines the decay of Process S	Optimized for each animal and derivation
rs	Rise rate of S	Determines the increase of Process S	Optimized for each animal and derivation
S_0_	Initial level of S at the beginning of the simulation		Adjusted for each animal and derivation
S_U_	Upper asymptote of S		Optimized for each animal and derivation

*The time unit used in the simulations is the time step ts = 4 s. rc, fc_R_, fc_W_, g_c_, r_s_ are expressed in ts^−1^; t_a_, t_p_, t_aw_, t_pw_ are expressed in ts; SWA_L_, SWA_0_, S_0_, S_U_ are expressed as a percentage of mean SWA over NREMS in baseline (regular wheel and complex wheel groups: 48 hr of baseline, enforced/exploratory wakefulness group: 24 hr of baseline).

SWA = slow-wave activity; NREMS = nonrapid eye movement sleep; REMS = rapid eye movement sleep.

Similarly to the original 1993 model, and to reduce computational time, we chose to optimize only a few parameters. Since some of the parameters are interdependent, we decided to fix first those parameters that could be evaluated by a visual inspection and had little influence on the error term, such as the fall constants, which were estimated by evaluating the slope of the decrease of SWA at the transition from NREMS to REMS/wake. We then allowed the optimization to adjust only a subset of remaining parameters (Table 1, see below for more details on those parameters which were chosen for automated optimization). As a rigorous sensitivity analysis sometimes led to aberrant results (the error is based on one mean value per NREMS episode, which is not suited to evaluate parameters determining the fine time course of SWA within NREMS episodes, such as fc_R_, fc_W_, and rc), for some parameter values (fc_W_, SWA_L_, ta/taw, tp/tpw), the final choice was based on visual inspection. By “visual inspection,” we mean that at least three different observers went through a zoomed-in version of a 48 hr simulation (such as in Supplementary Figure S2b) and evaluated whether the variations of SWA matched empirical levels well (timing of the increase/fall and maximum and minimum levels reached). In the first elaborated version of the two-process model [44], the constants fc_R_ and fc_W_ were introduced and governed the fall rate of SWA at the transition from NREMS to REMS and to wake, respectively. In this original paper, using human data, fc_W_ was approximately equal to 5*fc_R_. In the present version of the model, using mouse datasets, these two parameters were found to give the best results when their values were in a similar range (approximately 0.2 ts^−1^). By visual inspection, the variations induced by small variations of fc_W_ were almost nondetectable, which is why a value of 0.2 was applied. For fc_R_, however, the value chosen had a slightly bigger impact on the overall fit of the simulation to the data at NREMS to REMS transitions, and thus, we allowed the value to vary between 0.1, 0.2, and 0.3 depending on the animal or the derivation. SWA_L_ was fixed to 20% of SWA mean. The value of SWA_L_ does not contribute significantly to the error as it is an arbitrary value at which SWA levels stay during wake and REMS. After exploring various possibilities, and with the aim to set the same value for all animals, a value of 20% was kept as it was a good average of SWA levels in wake across animals. Starting values of rs, gc, S_U_ and S_0_ were derived from Franken et al. [59]. rs, gc, and S_U_ were then optimized, as described below, whereas S_0_ was chosen as equal to the mean value of SWA over the very first NREMS episode longer than 3 min (45 ts) recorded in 48 hr. SWA_0_ was also chosen individually for each animal and derivation as equal to the starting value of empirical SWA (i.e. at the beginning of the first light phase). t_a_, t_p_, t_aw_, and t_pw_ were evaluated by estimating the time taken by SWA to increase (respectively, decrease) before (respectively, after) a NREMS episode preceded (respectively, followed) by a REMS (for t_a_ and t_p_) or wake (for t_aw_ and t_pw_) episode (Table 1). Finally, rc was initially taken as equal to the average increase rate of SWA at the beginning of NREMS episodes following wake; we then optimized rc alongside gc, S_U_, and rs, but as its impact on the solution was small—while slowing the optimization process down—we chose to try multiple arbitrary values ranging from 0.2 to 1.5. After visual inspection, a value of 0.5 was chosen, which revealed the best fit in most animals.

### Optimization/estimation process of the parameters

We chose an optimization approach similar to the one used in the original elaborated version of the two-process model [44] and added an additional step to test the stability of the solution obtained at the end of the optimization (see Step-by-step optimization approach, and Supplementary Figure S3b). Using custom-made scripts, the parameters gc, rs, and S_U_ were optimized by minimizing the squared error (function *fminsearch*) between simulated and empirical SWA of NREMS episodes longer than 1 min. The time course of empirical SWA was smoothed with a moving median filter (moving window of *n* = 35 ts), as shown in Supplementary Figure S3a.

The squared error between simulation and data was calculated as follows: for a given *i*^th^ NREMS episode occurring in the light period, a mean SWA value was calculated from both the empirical data (m_empirical_(i)) and the simulated data (m_simulation_(i)). The difference between those two means was calculated: d(i) = m_empirical_(i) − m_simulation_(i). Those differences were squared and added over all NREMS episodes occurring in the light phase to give the following equation:

$$\text{M}(\text{light})=\overset{n}{\underset{i=1}{\sum }}\frac{d{\left(i\right)}^{2}}{n}$$

The same was done in the dark phase to generate M(dark). The overall squared error was then simply

$$Err=\;\frac{M\left(light\right)+M(dark)}{2}$$

The goal of the optimization was to minimize this squared error (i.e. to minimize the discrepancy between empirical and simulated data). Note that with this procedure, the time course of SWA within NREMS episodes was not fitted, as only mean SWA per episode was considered.

The approach of differentiating the light and dark periods—before averaging them together—aimed to avoid a bias in the optimization process, given that in mice there are naturally more NREMS episodes occurring in the light phase than in the dark phase (number of NREMS episodes over two light periods (2 × 12 hr): 155 ± 20 (mean ± SD); over two dark periods: 49 ± 22. Values calculated from the RW group). On the other hand, taking an equal weighing of the light and dark phases means granting greater confidence to NREMS episodes happening in the dark phase, given that they are three times less numerous than in the light phase; this is a concession we chose to make as the main changes in SWA usually occur during the dark period.

### Step-by-step optimization approach

The starting values for the three parameters gc, rs, and S_U_ were initially derived from Franken et al. [59], and after a few trials, a reasonable range of starting values was chosen and the following method was observed for each animal (Supplementary Figure S3b):

(1) A first step of nine optimizations combining three different starting values of gc with three different values of rs was performed individually for each animal and derivation. The starting value for S_U_ was kept the same for these nine optimizations. Those starting values were as follows:
  • In the frontal derivation: gc(0) = 0.0005, 0.0010, 0.0020 ts^−1^; rs(0) = 0.0001, 0.0002, 0.0004 ts^−1^; S_U_(0) = 400%.
  • In the occipital derivation: gc(0) = 0.0002, 0.0004, 0.0008 ts^−1^; rs(0) = 0.0001, 0.0002, 0.0004 ts^−1^; S_U_(0) = 450%.
(2) From the results of this first set of optimizations, the parameters yielding the smallest squared error were chosen as the starting values of a new run of nine optimizations, with the following rules (Supplementary Figure S3b):
  • gc(0) = gc value yielding best result − 0.0002 ts^−1^;
gc value yielding best result ts^−1^;
gc value yielding best result + 0.0002 ts^−1^;
  • rs(0) = rs value yielding best result − 0.00002 ts^−1^;
rs value yielding best result ts^−1^;
rs value yielding best result + 0.00002 ts^−1^;
  • S_U_(0) = S_U_ value yielding best result, for all nine [gc(0); rs(0)] combinations.

This means that for each animal and derivation, the set of starting values for the second set of optimizations was unique (unless two animals showed the smallest squared error for the same set of values). At the end of this second set of optimizations, the variation in the squared errors obtained was <10% (i.e. the ratio (highest squared error − smallest squared error)/smallest squared error < 0.10), which was considered satisfactory, as it showed a relative stability of the solutions obtained. No further step was performed and the values kept were those that yielded the smallest squared error in this second set of optimizations (Supplementary Figure S3b).

(3) Final *simulations* (as opposed to *optimizations*) using those optimized values of gc, rs, and S_U_ were obtained in each animal (and derivation) and are the simulated datasets referred to in this paper. Simulations used the exact same set of equations (1)−(4) as the optimizations and therefore also make use of the trigger functions REMT and WT. Parameter values used are those mentioned in Table 1, and for gc, rs, and S_U_, the ones determined from the optimization process described above. It is important to note that parameters were optimized against BL recordings and these BL values were kept to predict SWA and Process S levels in EW and CW recordings, in order to look at how waking behavior could then affect the adequacy of the model’s predictions.

### Calculation of the error to evaluate how well the model predicts the data

To compare empirical and simulated SWA, we used both an “absolute error” and an “error.” The absolute error consisted in taking the mean of the absolute differences d(i) described above over a given period of interest. The error is the mean of those differences (without taking their absolute value); this may result in an important bias, however, as the individual differences may cancel each other, but gives an indication of whether the model over- or under-estimates the empirical data.

### Statistical analysis

All statistical analyses were performed using SPSS Statistics (IBM Corp., Release 23.0.0.2). Tests used and their results are reported within figure legends, or within the Results section for tests run on data not included in the figures.

## Results

### The intraepisodic dynamics of SWA in mice can be described using the elaborated model

**Figure 1.**
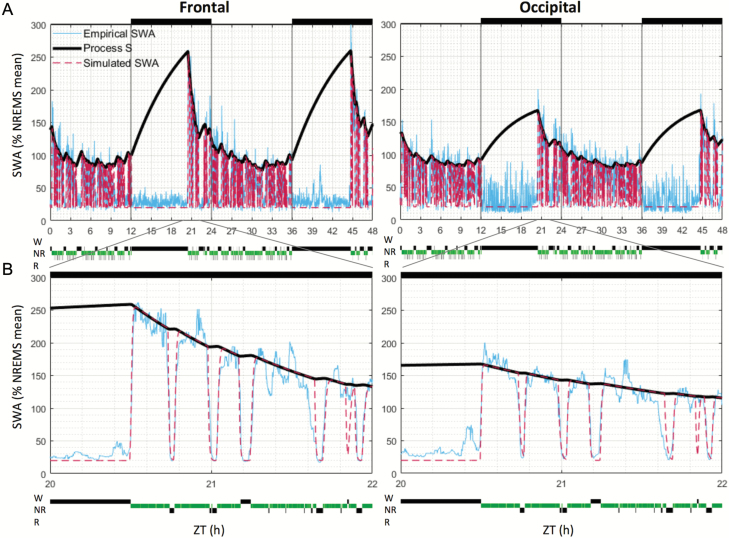
Simulated and empirical EEG SWA levels. EEG SWA levels are shown across 48 (A) and 2 hr (B) (from 20 to 22 hr) in one representative mouse, in the frontal (left panels) and occipital (right panels) derivations. Simulation results were plotted with one value per 4 s epoch, although the simulation solver was allowed to take steps of any size < 4 s (see Materials and Methods). SWA levels were normalized (expressed as percentage) against mean SWA over 48 hr in NREMS only. Light and dark phases are represented at the top of the figures (black bars = dark phase). NR = nonrapid eye movement sleep; R = rapid eye movement sleep; W = wake; ZT = zeitgeber time.

First, we investigated the performance of the model based on recordings obtained in a group of animals well habituated to regular running wheels (RW). As typical for mice kept in 12:12 hr light/dark conditions, the animals slept predominantly during the light period and were mostly awake during the dark period (Table 2, Supplementary Figure S4a). Extended wake bouts, likely encouraged by the access to a running wheel [67, 68], could be observed, and animals used their wheels voluntarily, spending an average 33 ± 7% (mean ± SEM) of the time awake running (Supplementary Text). As expected, the average EEG power spectra during NREMS, REMS, and waking showed characteristic differences (Supplementary Figure S4b). The time course of EEG SWA showed a declining trend across the light period and an increase after prolonged consolidated waking episodes (Figure 1). The initial levels of SWA were significantly higher in the frontal (Fro) derivation compared with the occipital (Occ) derivation, as has been described previously [49, 52, 69].

**Table 2.** Percentage of time spent in different vigilance states in the RW condition across 12 hr light and dark periods

	Nonrapid eye movement sleep	Rapid eye movement sleep	Wake	Brief awakenings
Day1-Light	60 ± 1%	11 ± 1%	25 ± 1%	4 ± 1%
Day1-Dark	17 ± 2%	2 ± 1%	79 ± 3%	1 ± 1%
Day2-Light	60 ± 1%	11 ± 1%	25 ± 2%	4 ± 1%
Day2-Dark	19 ± 3%	2 ± 1%	78 ± 4%	1 ± 1%

Values expressed as mean ± SEM. Brief awakenings are characterized by movement (electromyogram activity) happening during sleep for no more than 20 s (5 ts).

**Figure 2.**
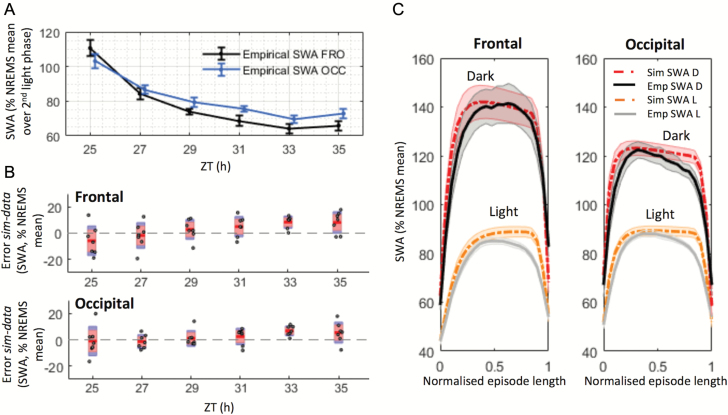
A close fit was obtained between empirical and simulated SWA levels. **(A)** The time course of empirical NREMS SWA levels in the light phase of the second day of RW condition in the frontal (FRO) and occipital (OCC) derivations. *n* = 7; mean ± SEM. 2 hr values of SWA were normalized against the mean over the second light phase only (see Materials and Methods). (**B**) 2 hr values of the difference between simulated and empirical SWA in the light phase of the second day of RW condition. Only SWA values in NREMS episodes lasting longer than 1 min were included in this analysis. Red area: 95% CI, blue area: 1 SD. Dots represent individual animals. The significance of the results was assessed with an ANOVA for repeated measures (factors “derivation” and “time point”), which revealed a significant main effect of time point (*F*(5,30) = 5.518, *p* ≤ 0.001) and a significant interaction derivation*time point (*F*(5,30) = 5.49, *p* ≤ 0.001). (**C**) Average empirical and simulated SWA levels during NREMS episodes, in light (L) and dark (D) periods. Only NREMS episodes lasting >3 min were included (average length = 7.9 ± 3.9 min, mean ± SD). Individual episodes’ lengths were normalized to an arbitrary length of 1, to allow for averaging between episodes. Note that SWA is higher in the dark than in the light period. *n* = 7, mean ± SEM. ZT = zeitgeber time.

To test whether the dynamics of SWA can be described by the model based on the 24 hr distribution of waking and sleep, we applied an “elaborated” version of the two-process model [44]. This version of the model accounts for the declining trend of SWA during sleep (as in the previous versions of the model), but also for the variations of SWA within successive NREMS episodes [44]. Figure 1, A and B depict SWA and Process S for an individual representative mouse. A good fit was apparent, both during periods with low SWA and during early sleep after prolonged waking bouts, when SWA levels are increased for both derivations. The empirical and simulated SWA values were similar across 24 hr, and the time courses of empirical and simulated SWA during NREMS fitted closely during both individual NREMS episodes (Figure 2C) and overall during the light periods, when mice are predominantly asleep (Figure 2B). We should point out, however, that as SWA decreased gradually throughout the light period (Figure 2A), the error between data and simulation also showed a weak increasing trend, mainly in the frontal derivation (Figure 2B).

Since SWA does show distinct region-specific dynamics on a time scale of minutes, where both long-term history and immediate preceding state play a role [60], we next investigated further the performance of the model within individual NREMS episodes (Figure 2C; lengths of NREMS episodes included = 7.9 ± 3.9 min, mean ± SD). Note that in Figure 2C, the dynamics of individual NREMS episodes (only NREMS episodes longer than 3 min were included and averaged) are represented, showing the build-up and decrease of SWA levels at the beginning and end of a NREMS episode. The two essential constants, which affect the rate at which SWA levels decrease at the transitions from NREMS to wake or REMS, are fc_W_ and fc_R_, respectively (Table 1). The higher the value of fc_W_ and fc_R_, the steeper is the decrease of simulated SWA levels at state transitions. Consistent with previous studies [43, 70, 71], we observed that SWA decreases sharply at the transitions from NREMS to wake or to REMS and increases at the transition from REMS or wake to NREMS (Figure 2C). This time course was similar in both derivations (Supplementary Material). The simulation followed these variations closely, although occasional mismatches with SWA levels could be observed. Relatively minor discrepancies were apparent between the empirical and simulated SWA towards the end of NREMS episodes immediately preceding REMS (Figures 1B and 2C). This may be related to the intrusion of spindle-activity and an instatement of mixed states, which increases within the light period in mice [71–74].

**Figure 3.**
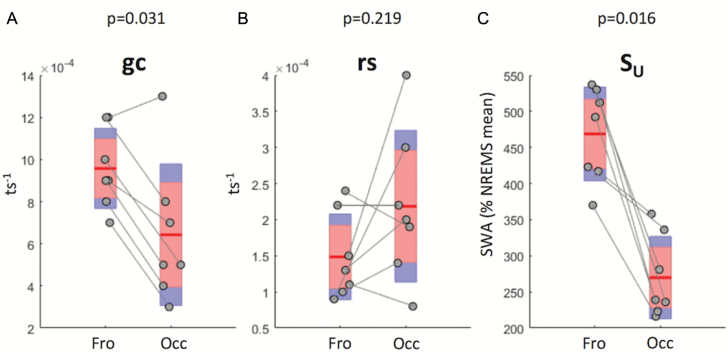
Values retained for the three optimized parameters of Process S. (**A–C**) Mean (red line) and individual animal values (grey circles) of the parameters gc, rs, S_U_ in the frontal (Fro) and occipital (Occ) derivations in the regular-wheel group. ts = 4 s; *n* = 7; red area: 95% CI, blue area = 1 SD. The significance of the difference in parameter values between derivations was assessed with a nonparametric Wilcoxon signed-rank test; *p*-values are indicated above each plot. SWA = slow-wave activity; NREMS = nonrapid eye movement sleep.

Since absolute EEG power levels, spectral composition, and the dynamics of SWA differ substantially between cortical regions [49, 52, 69, 75], we then addressed whether the parameters describing the dynamics of Process S also show regional differences. To this end, we performed optimizations for three essential parameters of Process S: its gain constant gc, rise rate rs, and upper asymptote S_U_ (see Materials and Methods) separately for the frontal and the occipital derivation (Table 1). Interestingly, we observed that gc, which affects the rate of decrease of Process S depending on SWA levels, was significantly higher in the frontal derivation (Figure 3A). Interestingly, rs, which determines the build-up of Process S, was similar in the two derivations (Figure 3B), whereas S_U_, the upper asymptote, was also significantly higher in the frontal derivation (Figure 3C). These results were conserved when pooling data from the RW and EW groups (Supplementary Figure S5) and suggest that both sleep–wake history and cortical region are the major intrinsic factors which determine the dynamics of Process S.

### The model’s performance is similar in the light and the dark periods

**Figure 4.**
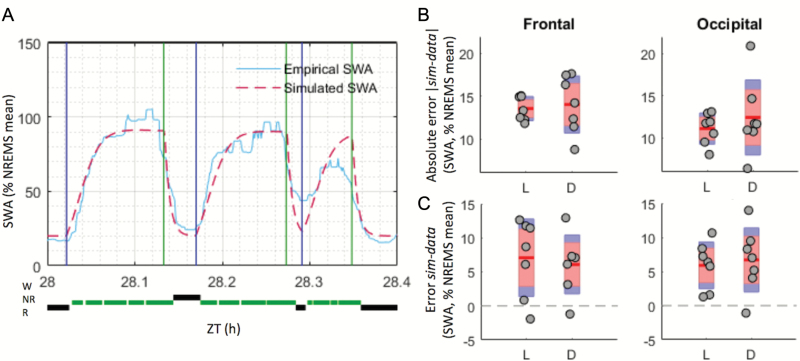
The SWA simulation fits empirical data similarly well in the light (L) and dark (D) phases. (**A**) Example of how the difference between mean values of empirical and simulated SWA levels (plotted with one value per 4 s epoch) were taken for each NREMS episode, before being averaged over L or D phases (see Materials and Methods). Beginning and end of NREMS episodes (i.e. where each simulated and empirical SWA means are calculated) are indicated, respectively, with blue and green vertical lines. For each NREMS episode (delimited by a blue and green line), one average SWA value was computed, as required by the optimization process chosen. Here, only 24 min from one animal are shown for clarity. (**B**) Mean (red line) and individual (grey circles) absolute differences between simulated and empirical SWA levels in the RW group. Values calculated over NREMS episodes lasting longer than 1 min; *n* = 7. Red area: 95% CI, blue area: 1 SD. Significance was assessed with a two-way ANOVA for repeated-measures, factors “derivation” and “light phase.” No significant main effect or interaction were found (derivation, *p* = 0.056; light phase, *p* = 0.529; interaction, *p* = 0.488). (**C**) As in (B), but the error (and not absolute error) value of the difference d = SWA_simulation_ – SWA_empirical_ is kept, allowing to see whether the model over- (positive difference) or under-estimated the empirical data. No significant main effect or interaction was found (derivation, *p* = 0.873; light phase, *p* = 0.956; interaction, *p* = 0.136). NR = NREMS; R = rapid eye movement sleep; sim = simulation; W = wake; ZT= zeitgeber time.

The time of day and lighting conditions are major extrinsic factors, which influence sleep–wake amount and quality across 24 hr. Is the model performance influenced by these factors? Importantly, as mice have much more waking time during the dark period (D), the squared error term could be biased by the larger contribution of the light period (L) towards NREMS. Furthermore, overall SWA was substantially higher during the dark period compared with the light phase (Figure 2C). To account for this potential confound, we calculated the mean squared error separately for the L and D periods during the optimization process and averaged the resulting values (see Materials and Methods, and Figure 4A). The simulation was found to fit empirical data similarly well during both L and D periods and in both derivations (Figure 4, B and C). To evaluate the fit between data and simulation further, we calculated the error in two ways (real and absolute values, see Materials and Methods). As shown by the error (Figure 4C), the model tended to slightly overestimate empirical average SWA levels (positive error) in both light and dark phases and in both derivations (Figure 4C), particularly towards the end of the light phase (Figure 2B; ANOVA, Fro: *F*(1,6) = 13.071, *p* = 0.011; Occ: *F*(1,6) = 26.667, *p* = 0.002), but no major difference between the L and the D periods was observed. Finally, we compared the performance of the model between the L and D periods by calculating SWA separately for NREMS episodes occurring during the L or D phase. Interestingly, despite a substantially higher overall level of SWA during the dark period, the performance of the model within individual episodes was similar across 24 hr (Figure 2C). However, as the amplitude of the absolute error seemed to be more variable in the dark phase across animals (Figure 4B), we tested the homogeneity of variances of the absolute error and error between L and D and across derivations by running paired-sample *t*-tests on the (absolute) differences between error-values and their group mean (as in a Levene’s test [76], but here the samples compared were not independent as errors in L and D were measured in the same group of animals). The value of the variance of the absolute error, but not error, was somewhat higher in D than in L in both derivations, but this was not statistically significant (Fro: *t*(6) = −1.957, *p* = 0.098; Occ: *t*(6) = −1.586, *p* = 0.164).

### The accuracy of the model’s prediction is robust irrespective of waking behavior

**Figure 5.**
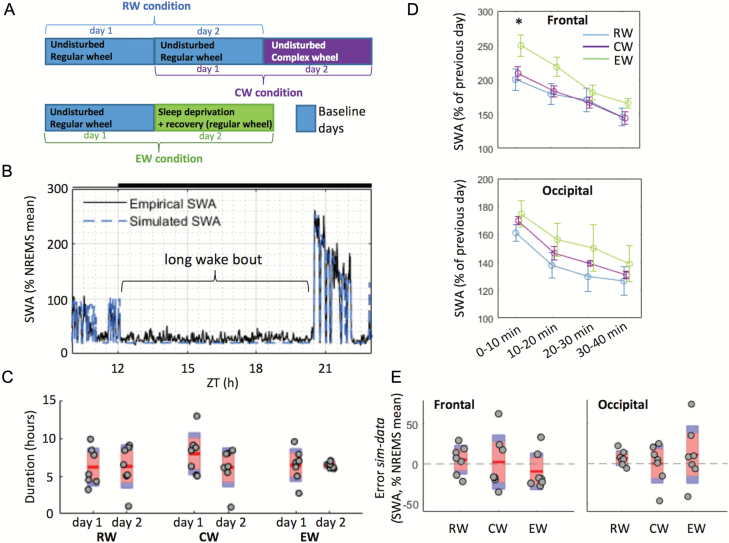
Impact of waking behaviour on SWA levels and on the accuracy of the model’s predictions following long wake bouts (Supplementary Material). **(A)** Definition of the 48 hr recordings used for each group: RW, CW, and EW. Blue rectangles: days used for optimization of the parameters; purple and green rectangles: days for which predictions were made. (**B**) Representation of a typical long wake bout in one animal from the RW group (plotted with one value per 4 s epoch). (**C**) Average “long wake bout” durations across three conditions (RW, CW, EW) in the first and second days. In each condition, *n* = 7; red area: 95% CI, blue area: 1 SD; grey circles represent individual animals. A mixed-design ANOVA (factors “condition” and “day”) revealed no significant main effects or interaction. (**D**) Time course of EEG SWA in NREMS during the 40 min following long wake bouts (10 min intervals) in three conditions. Mean ± SEM, *n* = 7 in each condition. SWA levels were normalized against mean SWA levels in NREMS during the previous 24 hr of baseline. An ANOVA followed by independent *t*-tests revealed a significantly higher value in the EW group in the frontal derivation in the first 10 min only (*t*(12) = −2.272, *p* = 0.042). (**E**) Average error between empirical data and simulations in three conditions in 40 min following long wake bouts (day 2). Only NREMS epochs were considered. For each condition, *n* = 7; red area: 95% CI, blue area: 1 SD; grey circles represent individual animals. The significance of the results was assessed using a mixed design two-way ANOVA (factors “derivation” and “condition”). There was no significant main effect of group or derivation, and no significant interaction group*derivation. sim = simulation; ZT = zeitgeber time.

We next investigated the impact of specific waking experience on SWA levels and on the fit between data and simulation following a prolonged bout of spontaneous or induced wakefulness (Figure 5, A and B). The direct comparison between the three conditions (RW, CW, and EW, see Figure 5A, and Materials and Methods) was enabled by the occurrence of an extended period of continuous wakefulness, which on average had a similar duration across groups and days (RW: 6.23 ± 2.58 hr, CW: 7.05 ± 2.74 hr, EW: 6.46 ± 1.50 hr, mean ± SD; Figure 5C and Supplementary Material for a detailed definition of long wake bouts). In all three conditions, the long wake bouts were invariably followed by an increase in SWA (Figure 5D), which was significantly higher after EW than in the RW and CW cohorts (*p* = 0.042), but only in the frontal derivation and immediately following wake. We hypothesized that since predominant waking behavior is different among RW, CW, and EW conditions, if this is an essential factor which affects SWA, then performance of the model should be different between the conditions. Interestingly, however, the model was robust to the preceding waking experience, and no statistically significant difference in the absolute error or error was observed between the groups (Figure 5E). However, as the CW and EW cohorts seemed more variable than the RW cohort, we performed a Levene’s test [76] to investigate differences in the homogeneity of variances. This revealed significantly different variances in the absolute difference *d* = |*simulation-data|* across the three conditions in the occipital derivation (Fro: *F*(2,18) = 2.442, *p* = 0.115; Occ: *F*(2,18) = 4.46, *p* = 0.027); further analysis revealed that the variance of the absolute difference (Occ) of the EW group was significantly higher (*F*(1,12) = 7.69, *p* = 0.017). As the absolute difference reflects the magnitude of the discrepancy between simulation and data (while the error gives an indication of the directionality of this discrepancy), this result shows that the model does not predict the data equally well in all animals in the minutes following total sleep deprivation (exploratory wakefulness, EW). To examine this aspect further, making use of a within-subject comparison, we compared the mean error over 40 min after long wake bouts occurring during the first (baseline) and second (EW) days specifically in the EW group. This analysis revealed only a minor difference in the error between days in the frontal derivation (Day1: 8.9 ± 2.3, Day2: −9.2 ± 8.7% mean SWA in NREMS, mean ± SEM, *F*(1,6) = 5.336, *p* = 0.060), and no difference was present in the occipital derivation (Day1: 9.1 ± 2.7, Day2: 11.5 ± 13.6% mean SWA in NREMS, mean ± SEM, *F*(1,6) = 0.034, *p* = 0.861). To rule out that this difference reflects a systematic difference in wake bout duration between conditions, we therefore asked whether the length of a long wake bout could have an impact on the fit between data and simulation directly following such a wake bout. No statistically significant correlation was found between long wake bout durations and the absolute error in the following 40 min (Supplementary Figure S6a). This suggests that if a sustained wake bout is present, the model accurately predicts the following SWA increase, irrespective of ongoing wake behavior or its specific duration.

### Wake analysis in RW, CW, and EW conditions

Since studies have shown that enforced wakefulness is associated with characteristic changes in the wake EEG spectra [75, 77], and, conversely, that wake quality may affect the dynamics of Process S [78], we next performed a detailed spectral analysis of the EEG during prolonged waking bouts. Waking was invariably characterized by high levels of θ-frequency activity in all three experimental conditions (Supplementary Figure S7a). However, an increase in θ-frequency (6–9 Hz) power was apparent in the CW group in the last hour of the prolonged wake bout, compared with the first hour (Supplementary Figure S7b). This suggests that waking combined with learning a new motor task could have had a different impact on some brain regions (the increase in θ power was significant in both derivations), compared with performing a stereotypical task such as running on a regular and familiar wheel. Interestingly, an analysis of running pattern, such as speed or time spent running (Supplementary Figure S1), revealed no significant difference between the baseline and complex-wheel day in the CW cohort. Notably, however, the introduction of a CW resulted in a shift of the peak of the running speed distribution towards lower paces (Supplementary Figure S1g), suggesting that the exposure of CW required an acquisition of a novel motor skill.

Additionally, although all three groups showed an increase in wake SWA in the last hour of the wake bout compared with the first hour, the EW group showed the highest increase in the occipital derivation (Supplementary Figure S7b—lower panel). These observations are consistent with previous studies showing that wake EEG undergoes changes across prolonged waking [77, 79–82]. However, there was no systematic relationship between the precise duration of the prolonged waking bouts and subsequent SWA levels in any of the three conditions (Supplementary Figure S6b). Furthermore, the value of the absolute error |simulation-data| in the initial 40 min interval after long wake bouts did not depend on the corresponding levels of empirical SWA (Supplementary Figure S8), suggesting that the model performance was good irrespective of the magnitude of SWA increase. Finally, there was no correlation between the percentage of time spent running on a wheel during long wake bouts and subsequent SWA levels or the performance of the model (Supplementary Figure S9).

## Discussion

### Performance of the model

Here, we applied for the first time an elaborated version of the two-process model to sleep recordings obtained in mice. The primary aims of this work were to assess whether the model could describe the dynamics of sleep in mice, and whether the time of day, lighting conditions, or specific waking behaviors influence the performance and predictions of the model. Furthermore, by using the EEG recorded from two anatomically and functionally distinct cortical regions, we formally addressed whether sleep homeostasis in mice is a global process or whether it has a local component. Our main result was that the elaborated model can be successfully used to simulate the dynamics of SWA in mice on time scales spanning from minutes to hours. Although some of the essential parameters differed between cortical areas, we found that the model was overall robust to extrinsic factors that are known to have a major influence on wake and sleep, such as the time of day or waking experience.

### Regional differences in Process S regulation

It is well known that SWA levels and dynamics differ across brain regions [49, 52, 69, 75]. Topographic differences in the dynamics of Process S in mice were highlighted for the first time in 2000 [52], and accordingly, our study showed differences between frontal and occipital areas in the regulation of Process S. Specifically, we report that the decay rate (or gain constant) of Process S is higher in the frontal derivation in mice, manifested as a faster decline of Process S in the anterior cortical area, which is consistent with human data [50, 51]. In 1998, it was suggested that recovery processes occur at specific locations in the brain and that SWA variations are closely controlled in a topographical way [83]. Our study, therefore, agrees with such observations, as Process S, which is intrinsically correlated to SWA levels, displays different dynamics between the frontal and occipital areas. Additionally, our findings show that EW induced a larger increase in SWA in the frontal derivation compared with the RW condition, an effect which is not observed in the occipital derivation (Figure 5D). A possible explanation for the topographical differences observed in the dynamics of Process S could be linked to prior waking activity. Indeed, it has been reported that sleep pressure, reflected in SWA levels, may build up at different rates in various areas depending on which brain regions have been mostly active during previous waking [53, 80, 84, 85]. It has also been shown that the increase of SWA following sleep deprivation is higher in the anterior prefrontal regions [8, 54, 82], suggesting again that there is a differential local build-up of sleep need. However, another likely explanation is that the difference in the dynamics of Process S between cortical areas is related to anatomical differences [86].

### Light-dark differences

The effect of lighting conditions was investigated here using two complementary approaches. First, we compared the error between the light and the dark phases during spontaneous undisturbed conditions. Second, we compared the performance of the model during the initial sleep after a prolonged waking bout terminating in the middle of either the dark or the light period. Although we found that the simulation fitted the data equally well in both L and D, the amplitude of the absolute error was more variable (although not significantly) in the dark phase across animals (Figure 4B). One possible interpretation for the fit between data and simulation being more variable across animals in D than in L is that there is generally a pronounced variability in sleep–wake distribution between individual animals during the dark phase, as the amount of sleep is lower and specifically NREMS episodes tend to be shorter. This finding is in agreement with Franken et al. [45], in which a better fit between data (recorded in rats) and simulation was obtained by using different time constants of the decrease of Process S in the light and dark periods. It cannot be excluded that there is a direct circadian influence—that the model does not currently tackle—which would lead to different rates of increase and decrease of Process S depending on the time of day. Indeed, one study [87] using recordings performed in rats kept in constant darkness also showed that the build-up rate of SWA changes with circadian time, thus showing that this is not caused by a direct influence of light, but rather by a circadian modulation of the SWA dynamics. Additionally, another study reported that circadian rhythmicity significantly influences slow-wave characteristics in humans [88], and a circadian influence was postulated earlier in simulations using the elaborated two-process model in humans [89]. It may be important to note that the circadian influence on the dynamics of SWA and Process S may be much stronger in some species than in others. For example, studies in humans [89] and rats [45, 87] obtained a better fit between data and simulation when allowing the time constants of the model to vary with the time of day, while this has so far not been proposed in mice. Indeed, the fit is usually satisfactory when keeping the constants unchanged across 24 hr [46, 59]. Accordingly, the performance of the model in our study was not markedly different between the initial sleep after a period of spontaneous waking during the dark period and after sleep deprivation during the light phase. It was possible to make a direct comparison since the mean duration of the main waking bout was similar between the conditions (Figure 5C, Supplementary Material). However, it cannot be excluded that not only the lighting conditions, but also the type of behavior is different between spontaneous wake in the dark period and sleep deprivation during the light phase.

### Impact of wake experiences

Our next hypothesis was that waking experience is an important factor responsible for instantaneous changes in Process S. If this prediction was correct, the model would overestimate the levels of “sleep need” if prior waking was mainly “quiet” or dominated by stereotypic behaviors, or conversely, underestimate sleep pressure if an animal is engaged in “demanding” activities, such as learning a novel motor skill or exploratory behavior. Although wake has long been considered as a homogeneous state, it cannot be excluded that waking dominated by automatic behaviors (such as regular wheel-running) may lead to a slower accumulation of sleep need than nonautomatic activities [62, 90]. To test the above hypothesis, we used data recorded in animals which had access to CWs during the dark period. Prior to the exposure to the CW, these animals were well adapted to run on a RW, which they used extensively. However, the use of a CW requires a different pattern of running, which is associated with sensorimotor learning. Surprisingly, we found that the introduction of a CW did not have a significant impact on how well the predictions of the model fitted the data, and the levels of SWA during subsequent sleep were not significantly different from corresponding values after RW running. It cannot be excluded that this task was not sufficiently demanding, especially as the animals had previously obtained extensive experience of running on a RW. However, the presence of a CW did trigger an increase in EEG power density in the θ range over the course of waking. Although the interpretation of this finding is not straightforward, it suggests that in some respects waking involving running on a CW was qualitatively different. This could reflect either a higher arousal level or learning processes, both of which have been associated with an increase in EEG θ-activity [62, 77, 79, 91]. At any rate, our results suggest that wake duration is likely the main factor that accounts for the dynamics of Process S, above and beyond the contribution of specific wake experiences.

Although it has generally been assumed that homeostatic pressure increases with time spent awake, a recent study proposed a reformulation of the sleep homeostasis model, whereby Process S would rise only during wakefulness dominated by high θ (5–10 Hz) activity [78]. Although this is an interesting possibility, an important potential caveat is that the occurrence of θ-rich waking may be related to an overall longer waking duration, which makes it difficult to disentangle the two factors. We argue that rather than Process S building specifically during θ-dominated waking, it is possible that other factors, which are manifested in more active waking behaviors, may in turn result in prolonged sustained wakefulness and therefore a robust build-up of sleep need. Additionally, it cannot be excluded that factors such as an elevation of brain temperature or metabolic rate during active waking may influence the rate at which sleep need increases [59, 92, 93].

### Limitations

One aim of the present study is to investigate the applicability of an elaborated version of the two-process model to mouse data. However, all analyses were performed using data recorded in a single strain (C57BL/6J). Given the variations already observed by Huber et al. [46], significant variations in the parameters—and the performance—of the model between different strains may be expected. Similarly, we did not use recordings performed in female mice, and the possibility remains that there are gender differences in the effects of extrinsic factors on sleep homeostasis. In addition, only two areas of the neocortex were investigated (motor and visual cortex) and the study of the topographical variations of SWA dynamics could benefit in the future from recordings performed in other regions. Since the main finding of this study—that the model appears robust to some important extrinsic factors—could be considered as a negative result, the possibility remains that adding further animals could reveal significant differences. However, we find this possibility unlikely since the number of animals we used was in line with previous studies [45, 46, 52, 56, 58, 59] and no systematic trends were apparent among individual mice.

Finally, we did not find any significant impact of varying waking behaviors on the performance of the model. This does not exclude the possibility that sleep pressure may build up or dissipate at different rates with or without access to a RW or CW, but that our modeling approach was not sensitive enough, nor does it exclude that other waking experiences may have had a stronger influence on the dynamics of Process S. For instance, more stressful activities, which have been shown to enhance sleep intensity in the following sleep episode [94], may have resulted in an increased error between data and simulation when using parameters optimized on baseline. Additionally, to test the possibility that a stereotypic waking behavior such as wheel-running reduces the build-up of Process S, a preferable approach would have been to compare to a nonrunning waking behavior (e.g. sleep deprivation by handling or spontaneous waking without access to a running-wheel during the dark period), instead of the approach chosen here where mice had always access to a running wheel, and where therefore the impact of the “nature” of the wheel (RW versus CW) was analyzed.

### Conclusions

The striking robustness of the model that we observed across a variety of conditions may reveal a fundamental—although yet to be fully defined—aspect of sleep homeostasis. It appears that as long as a mouse is awake, Process S builds up, and if the mouse is asleep, it decreases, regardless of circadian time, light or waking behavior; the only difference that was consistently observed was between cortical regions. We therefore hypothesize that initiation, maintenance, and termination of wake and sleep states are largely independent of sleep–wake history, and instead primarily determined by ecological and extrinsic factors, such as circadian time or lighting conditions, and vital needs, such as hunger. This aspect of sleep regulation is thus likely distinct from the system that keeps track of time spent awake or asleep. Although this hypothesis remains to be tested empirically, our findings, supported by the modeling approach, are consistent with numerous observations that homeostatic sleep drive can be overridden by a variety of factors, and sometimes for extended periods of time, such as during migration [95, 96] or when food is restricted [97–99]. Furthermore, this scenario is compatible with the view that sleep has features of a default state [100–104], which suggests that the necessity and the capacity to sustain wakefulness are the main target of regulation. Clearly, even if the biological necessity to remain awake is high, it is unlikely that sleep can be fully uncoupled from the circadian rest-activity rhythm for longer than a short period of time without deleterious consequences [105]. On the other hand, the continual interaction between intrinsic and extrinsic factors regulating sleep may be essential to allow most efficient sleep, both with respect to internal needs and taking into account ecological factors [106].

## Supplementary Material

Supplementary material is available at *SLEEP* online.

Supplementary Figure S1

Supplementary Figure S2

Supplementary Figure S3

Supplementary Figure S4

Supplementary Figure S5

Supplementary Figure S6

Supplementary Figure S7

Supplementary Figure S8

Supplementary Figure S9

Supplementary Text

Supplementary Figure Legends

## Funding

This work was supported by MRC NIRG (MR/L003635/1), BBSRC (BB/J014427/1, BB/K011847/1), Wellcome Trust Strategic Award (098461/Z/12/Z), John Fell OUP Research Fund Grant (131/032), Clarendon Scholarship (funded by the University of Oxford and Christ Church College), NSF grant 32003B_146643, and ESA Ariadna grant 4000114025/15/NL/LF/as.

## Notes

Work Performed: University of Oxford, United Kingdom
