# Supplementary figures and images for: Cortical region–specific sleep homeostasis in mice: effects of time of day and waking experience
Source: Sleep. 2018 Apr 25;41(7):zsy079. doi: 10.1093/sleep/zsy079 (PMC6047413; doi:10.1093/sleep/zsy079)

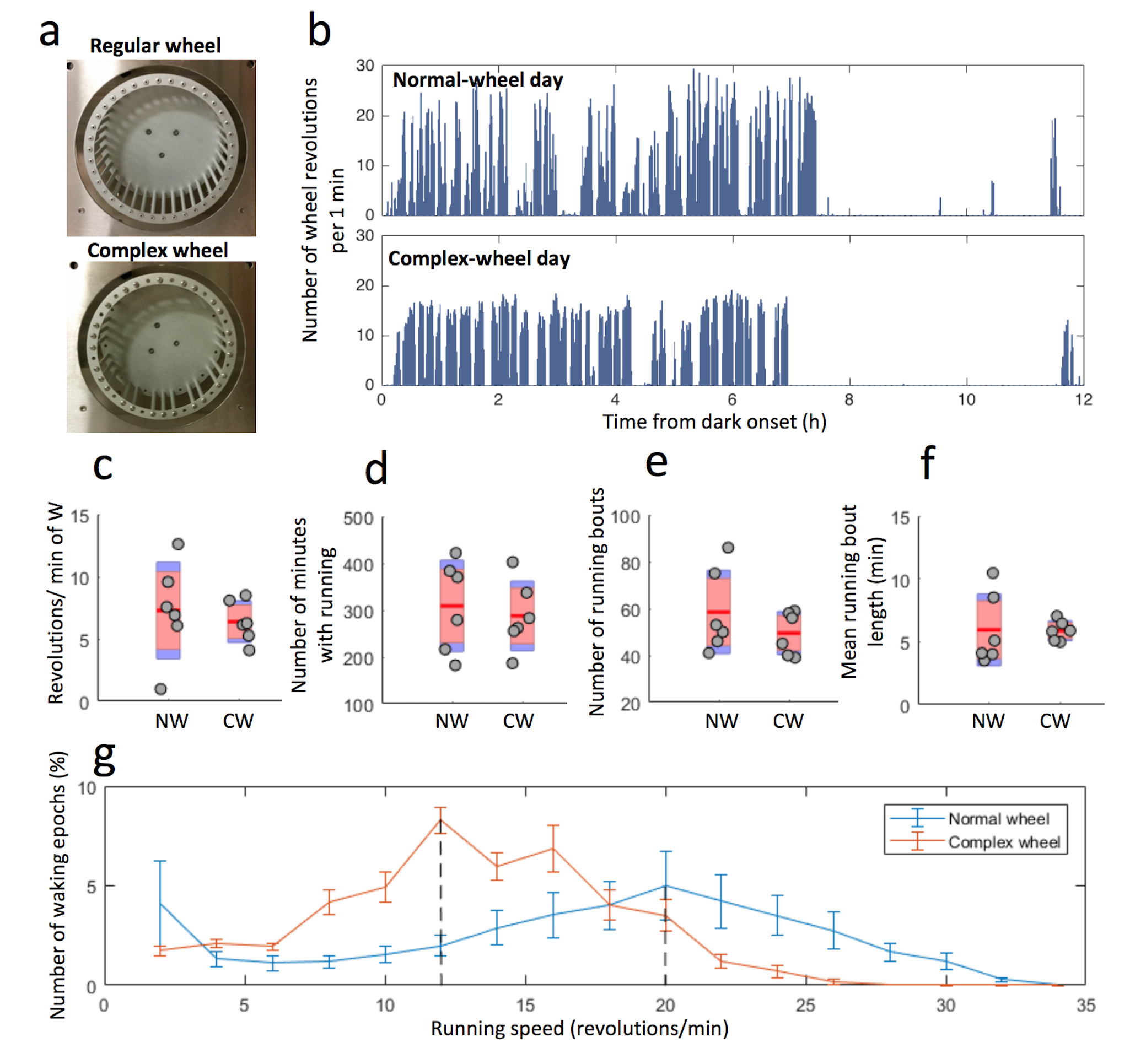

Supplement: Supplementary Figure S1 [file zsy079_suppl_figure_s1.png]

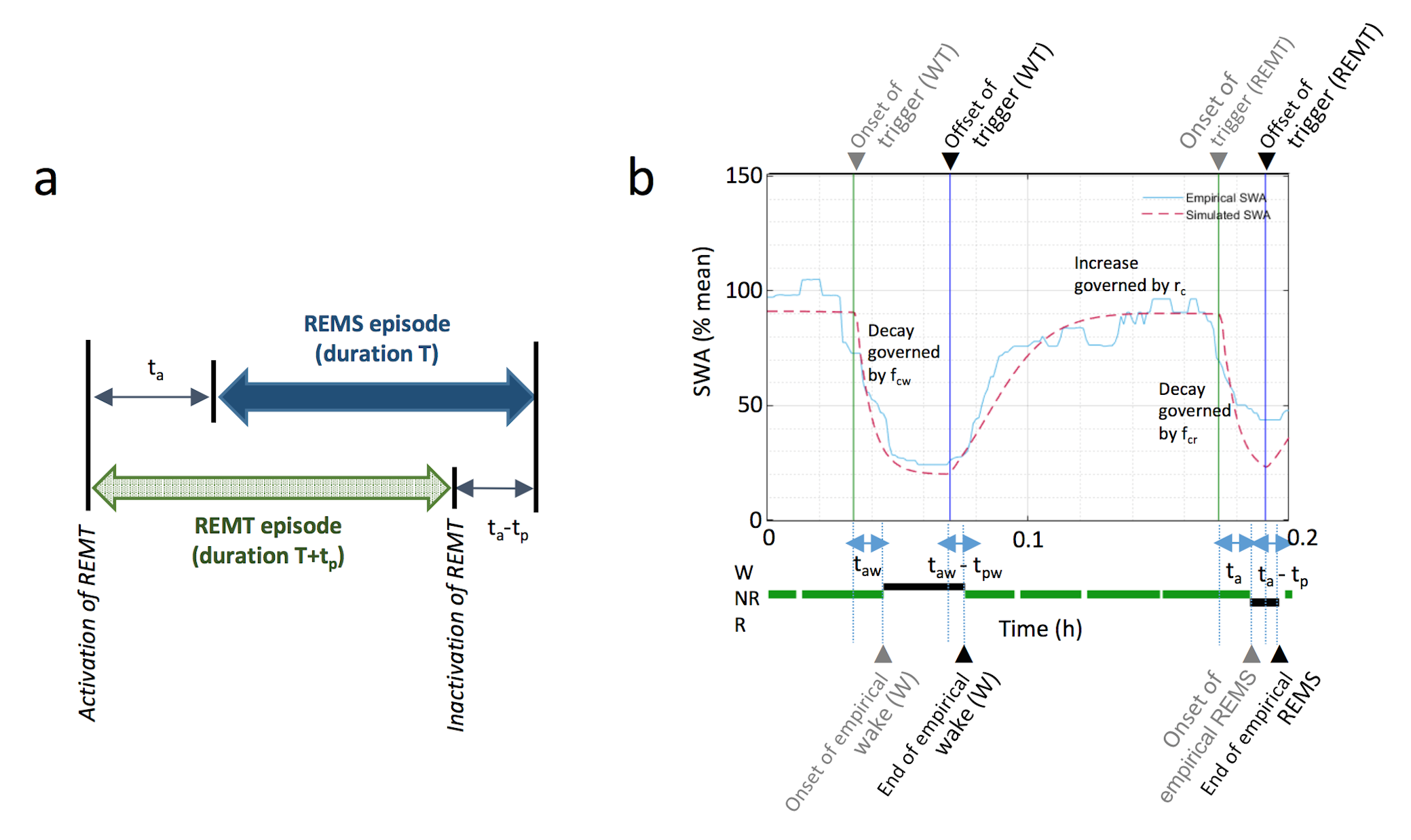

Supplement: Supplementary Figure S2 [file zsy079_suppl_figure_s2.png]

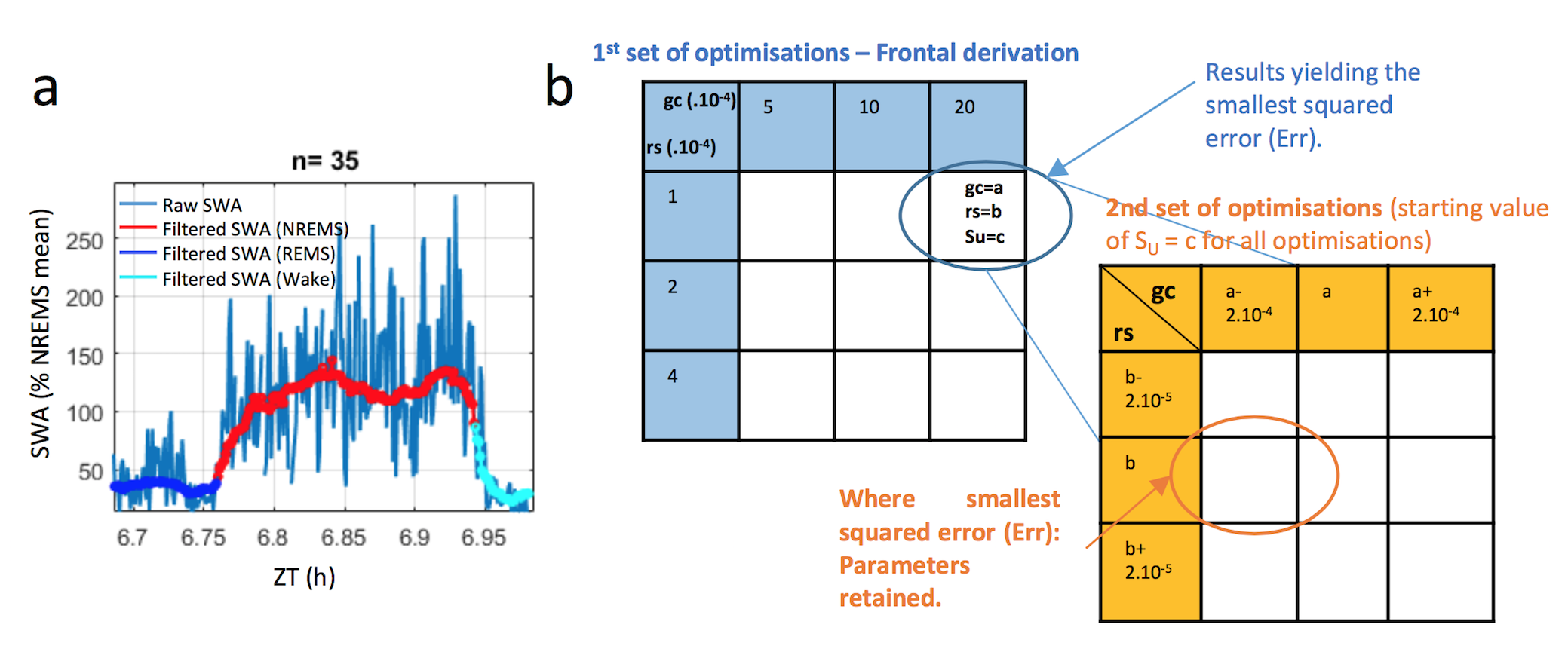

Supplement: Supplementary Figure S3 [file zsy079_suppl_figure_s3.png]

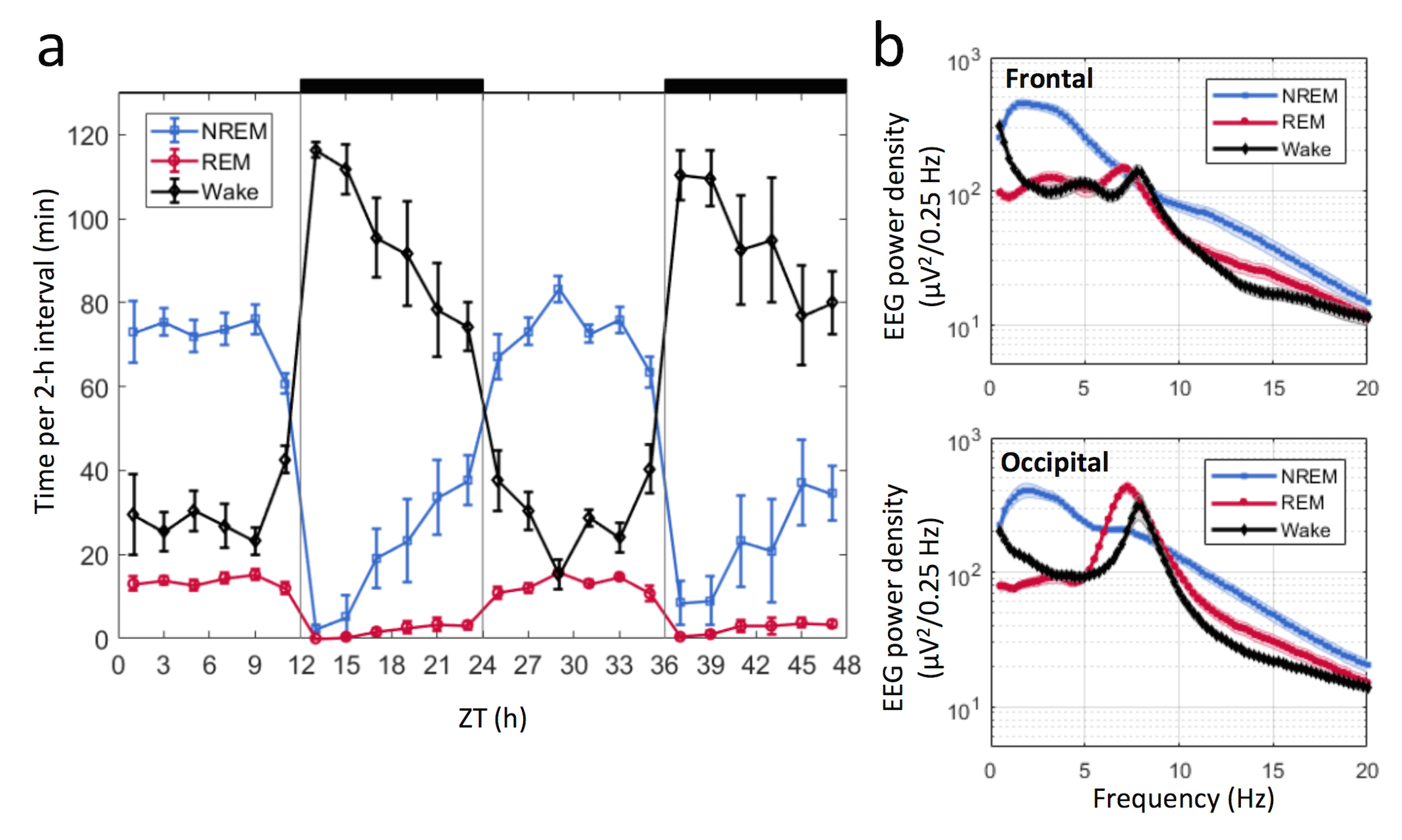

Supplement: Supplementary Figure S4 [file zsy079_suppl_figure_s4.png]

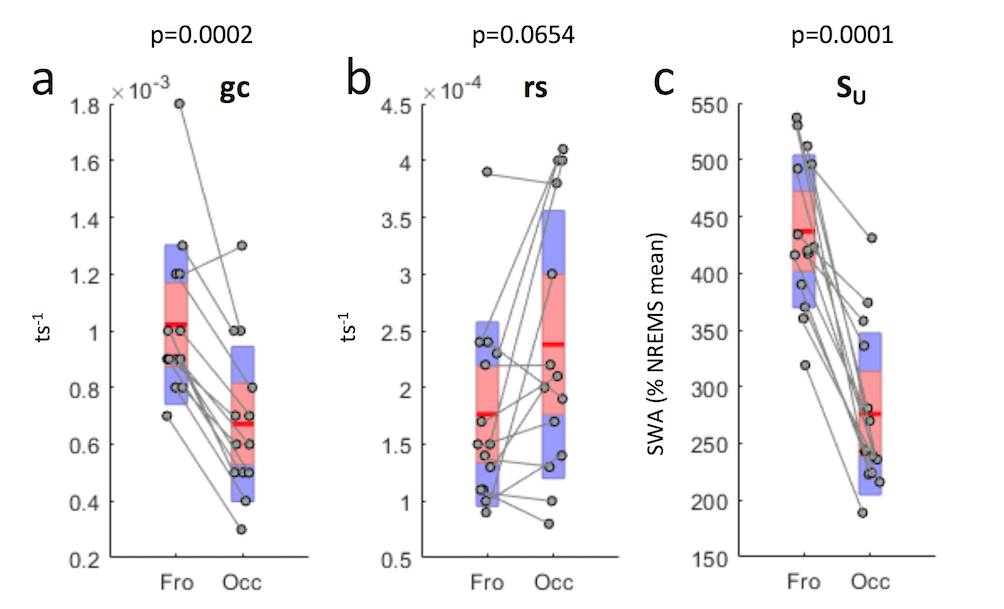

Supplement: Supplementary Figure S5 [file zsy079_suppl_figure_s5.png]

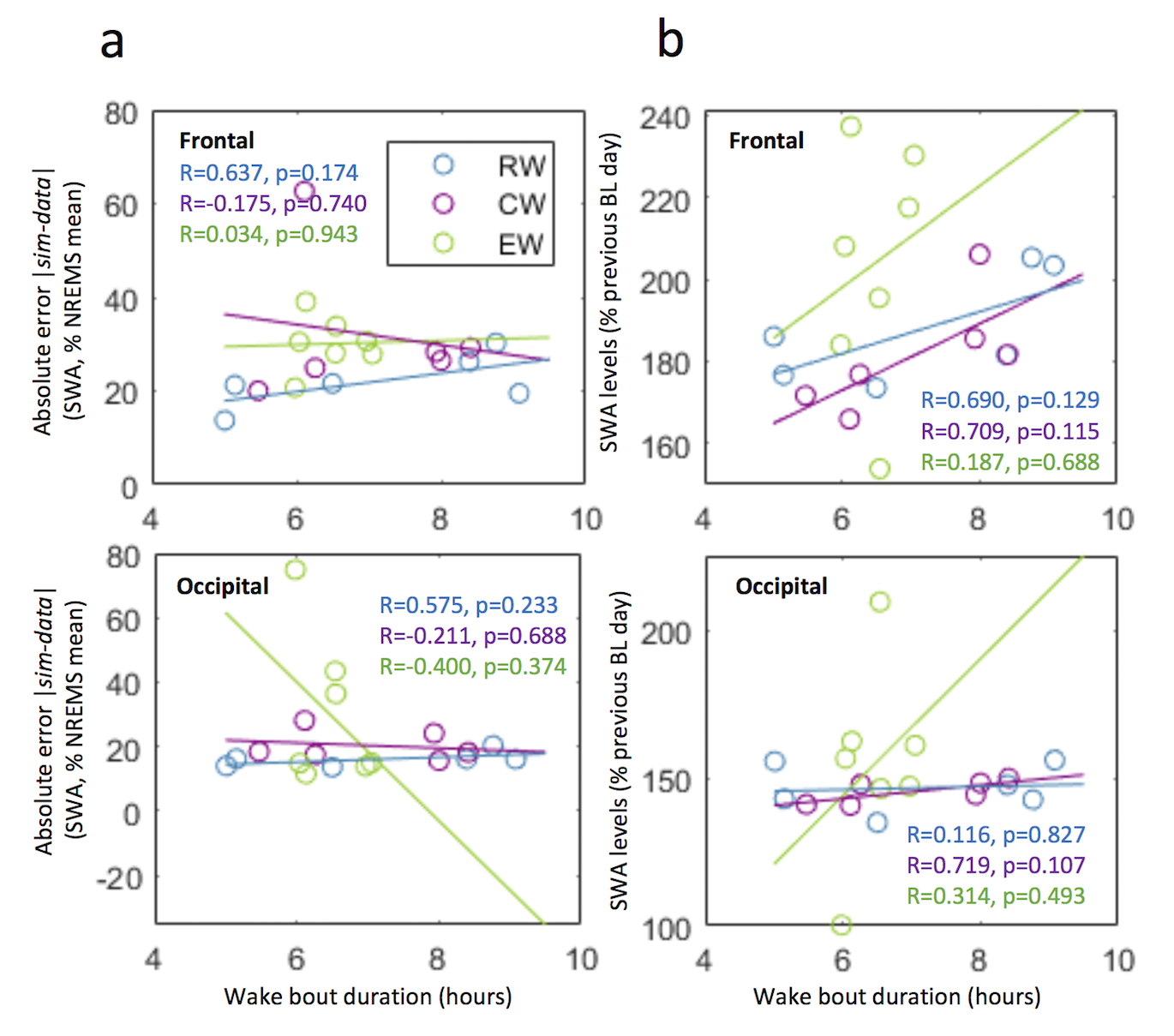

Supplement: Supplementary Figure S6 [file zsy079_suppl_figure_s6.png]

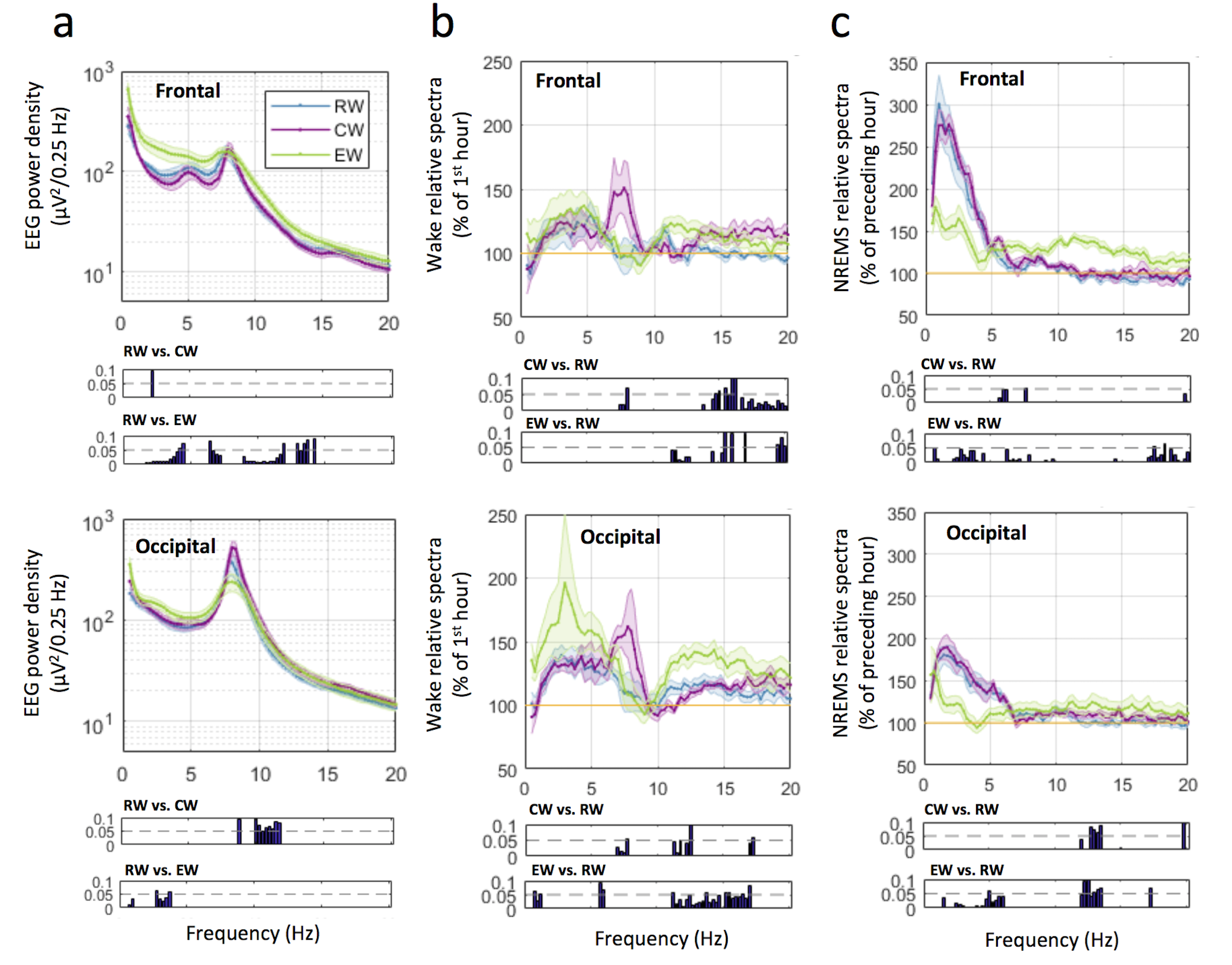

Supplement: Supplementary Figure S7 [file zsy079_suppl_figure_s7.png]

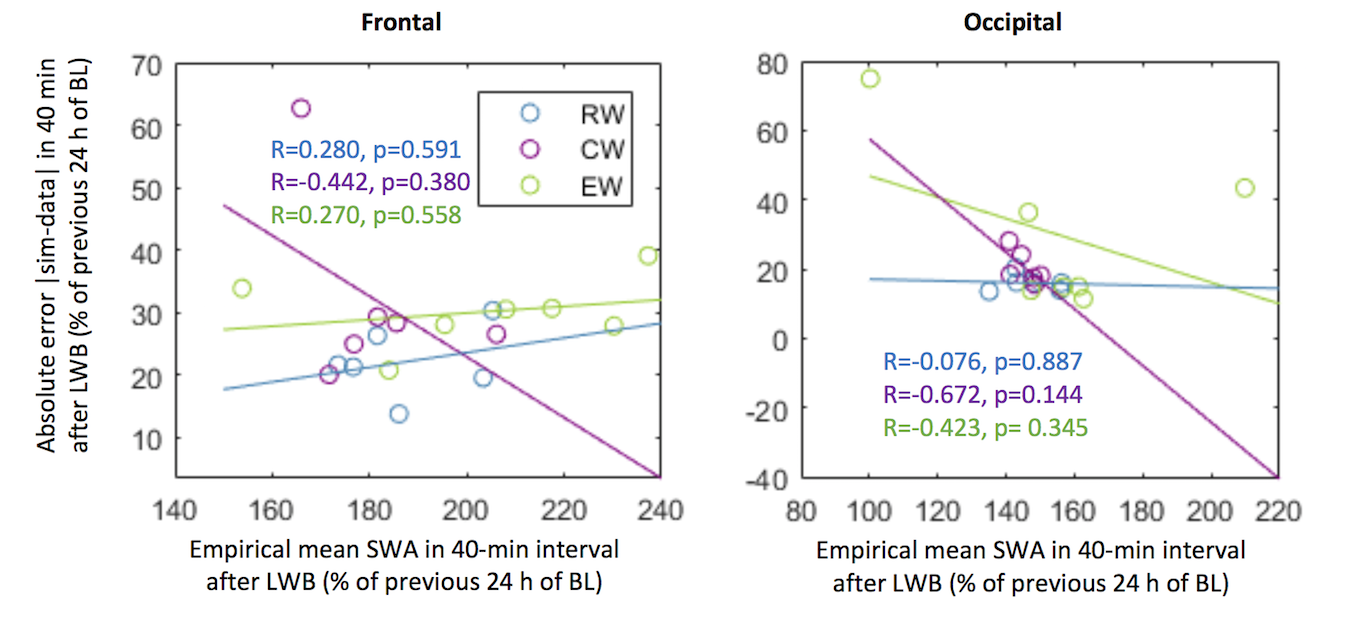

Supplement: Supplementary Figure S8 [file zsy079_suppl_figure_s8.png]

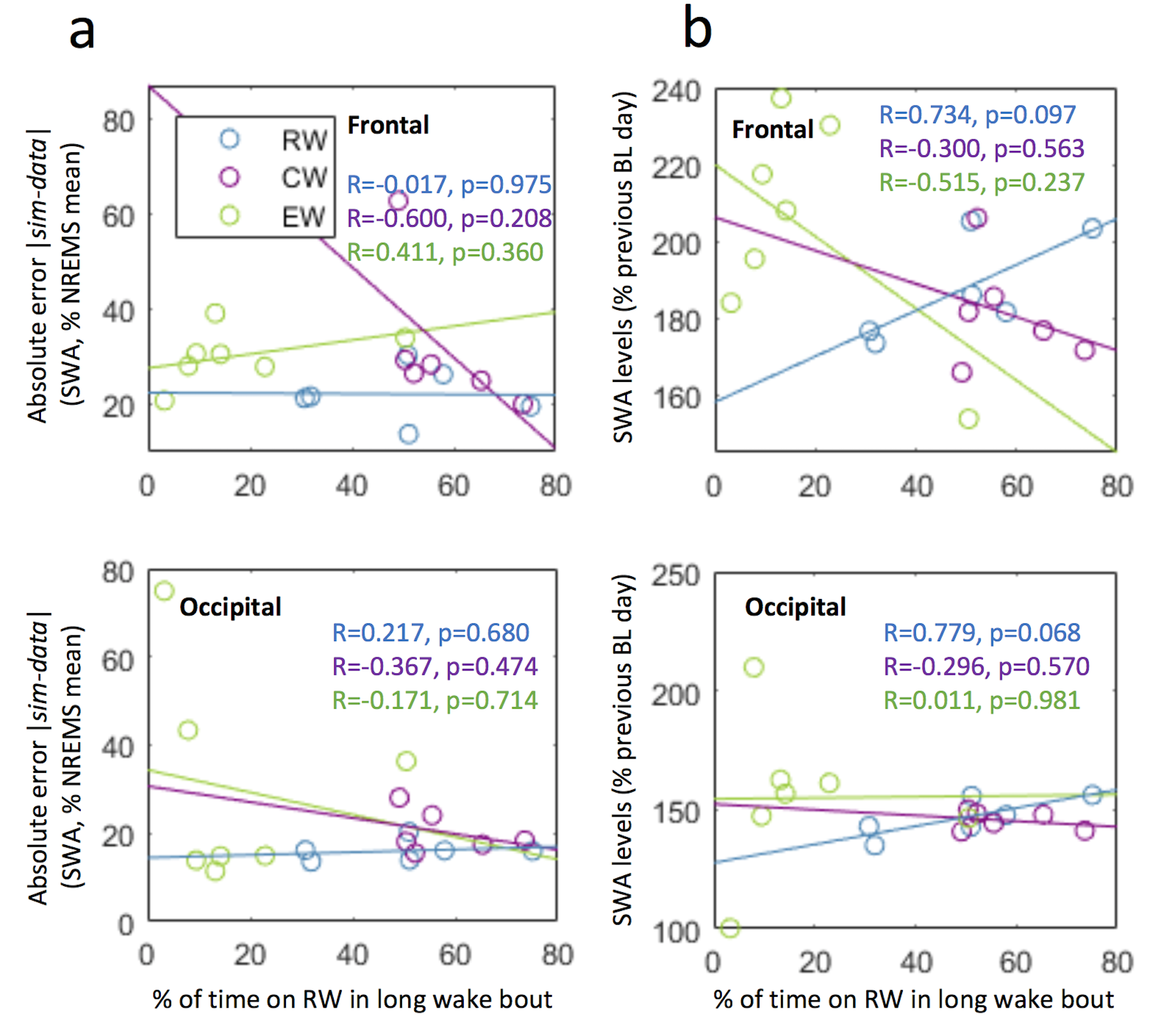

Supplement: Supplementary Figure S9 [file zsy079_suppl_figure_s9.png]
