# Supplementary material for: Cortical region–specific sleep homeostasis in mice: effects of time of day and waking experience
Source: Sleep. 2018 Apr 25;41(7):zsy079. doi: 10.1093/sleep/zsy079 (PMC6047413; doi:10.1093/sleep/zsy079)
Supplement: Supplementary Text [file zsy079_suppl_si_text.docx]

***SI Text.***

**Long waking bouts.** Only 25±1 % (mean ± SEM, n=7) of time was spent awake during the light period, while prolonged consolidated waking periods were apparent during the dark period, when the animals were awake 79±3 % of the time. In all but one individual animal, a long waking period starting around light-to-dark transition and lasting on average 6.2±1.0 h, could be observed, preceded and then followed by a consolidated period of sleep. The latency between dark onset and the beginning of the bout was less than 6 min (except in one animal for which it took 5 h for a consolidated wake bout to occur). The recordings were stable over the two-day period included in the analyses, and no difference between the two days in the amount of wake, NREMS and REMS was apparent (see Table 2).

The extended periods of wakefulness were induced by spontaneous stereotypic running (RW cohort), learning a novel motor skill (CW) and exploration of novel objects (EW cohort) respectively (Figure 5a-c). “Long wake bouts” were subsequently defined as the longest waking period for each animal on a given day. Notably, those long wake bouts did not significantly differ in length between the 3 groups and between the first and second day (Figure 5c). It is important to note that in the EW group, on the second day, the longest waking bout corresponded to the sleep deprivation period of 6 h and thus happened during the light phase (and not in the active dark phase as for other days and groups) and that variability in duration was thus the smallest. The fact that spontaneous long wake bouts in the RW and CW groups and during the first day of the EW group lasted around 6 h too, leading to no significant difference in long wake bout durations between groups and across days, was a welcome coincidence.

**Fall constants in humans and rodents.** It is interesting to note however, that in humans, for whom the transitions between different vigilance states occur overall less frequently over a 24-h period than in rodents, the fall constants differ significantly between REMS and wake^44^, while in rodents those two constants can be taken equal and still yield very good predictions of the model.
