# Supplementary material for: Cortical region–specific sleep homeostasis in mice: effects of time of day and waking experience
Source: Sleep. 2018 Apr 25;41(7):zsy079. doi: 10.1093/sleep/zsy079 (PMC6047413; doi:10.1093/sleep/zsy079)
Supplement: Supplementary Figure Legends [file zsy079_suppl_si_figure_legends.docx]

***SI Figure legends.***

**Supplementary figure S1.** *Patterns of running on a normal and complex wheel in the complex-wheel cohort.* **a)** Photos of a regular running wheel (left) and a complex running wheel (right). **b)** Bar plots of running speed (wheel revolutions/min) in each 4-s epoch across the dark period in the first day (access to a normal wheel - NW) and second day (complex wheel - CW) of one animal from the CW cohort. **c)** Average running-wheel revolutions per minute of waking in the dark period of the NW and CW days of the CW group. **d)** Total number of minutes with running activity recorded in the NW and CW days (dark periods only) of the CW group. **e)** Number of running bouts in the dark period of the NW and CW day of the CW group. Bouts were defined with a minimum duration of 16 s (4 x 4-s epochs); short interruptions of up to 16 s within a bout were permitted. **f)** Average running-bout lengths (expressed in minutes) in the dark period of the NW and CW day of the CW cohort. Running bouts were defined as in e). **c-f)** Red line: group mean, red area: 95-% CI, blue area=1 SD, grey circles represent individual animals. ANOVAs for repeated-measures revealed no significant differences between the NW and CW days in average speed of running (c), amount of running (d), running bout numbers (e) and running bout lengths (f). **g)** Distribution of running speed (expressed in revolutions/min) across waking epochs. Only epochs with an average running speed of at least 2 revolutions/min were plotted. Data was subdivided in 2 revolutions/min bins. The number of epochs within a given bin is expressed as a percentage of the total number of waking epochs. Error bars: mean ± SEM. Dotted lines highlight the running speed at the peak of each distribution curve. **c-g)** Only 6 animals of the CW cohort were used as one mouse did not run and was therefore excluded from analyses reported in panels b-g.

**Supplementary figure S2.** *Definition of rapid-eye movement sleep (REMS) and wake trigger functions.* **a)** As compared to a REMS episode, a rapid-eye movement trigger (REMT) episode starts t_a_ epochs earlier and last t_p_ epochs longer (with t_p_< t_a_); this means that the offset of REMT is t_a_-t_p_ epochs before REMS ends. This works identically for the wake trigger function (WT) in relation to wake episodes (with t_aw_ and t_pw_ replacing t_a_ and t_p_). **b)** Onsets (green vertical lines) and offsets (blue vertical lines) of WT and REMT in relation to empirical wake and REMS episodes (empirical states indicated in the hypnogram below the graph) are shown. The decay and decrease rates governing the variations of simulated slow-wave activity are indicated on the plot. NR= non-rapid eye movement sleep; R= REMS= rapid-eye movement sleep; REMT= rapid-eye movement sleep trigger; SWA= slow-wave activity; W= wake; WT= wake trigger.

**Supplementary figure S3.** *Illustrations of the smoothing of empirical data and of the optimisation process.* **a)** Example of smoothing empirical slow-wave activity (SWA, plotted on a 4-s epoch basis) with a moving median filter (n=35, i.e. 35 ts = 35 x 4 s, per moving window). Smoothing was performed continuously regardless of vigilance states (which are shown in different colours here for clarity). The mean value of the red segment would represent the mean empirical SWA levels for this given non-rapid eye movement sleep (NREMS) episode (see Methods). **b)** Diagram illustrating the optimisation process of the 3 parameters gc (gain constant of Process S), rs (rise rate of S) and S_U_ (upper asymptote of S) – see the Methods section for more details. To initialise the optimisation, 3 starting values for gc and rs were evaluated (Frontal: gc= 5, 10, 20 x10^-4^ ts^-1^; rs= 1, 2, 4 x10^-4^ ts^-1^ – Occipital: gc= 2, 4, 8 x10^-4^ ts^-1^; rs=1, 2, 4 x10^-4^ ts^-1^), where the orders of magnitude of these parameters were realistically chosen based on existing literature. Similarly, a good initial estimate of S_U_ was obtained from literature (Frontal: S_U_= 400 % – Occipital: S_U_=450 %), and this starting value was chosen for further optimisation with all 9 possible combinations of gc and rs (blue table – derivations treated separately). Starting from the values a, b and c of gc, rs and S_U_, which yielded the smallest squared error after optimisations of the first round, a second optimisation round was performed (orange table). During this optimisation round, small variations (gc: ±0.0002 ts^-1^; rs: ±0.00002 ts^-1^) around the values of a and b were evaluated and the final values used for each animal were those yielding here the smallest squared error. ZT= zeitgeber time.

**Supplementary figure S4.** *The distribution and EEG spectral power of vigilance states are as expected in C57BL/6J mice with access to a regular wheel (RW).* **a)** Time spent in each stage (wake, rapid eye movement sleep (REM), non-rapid eye movement sleep (NREM)) per 2-h interval. n=7 (RW condition); mean ± SEM. **b)** Mean EEG power density spectra of the different vigilance states in the frontal and occipital derivations. n=7 (RW condition); mean ± SEM. ZT= zeitgeber time.

**Supplementary figure S5.** *Values retained for the 3 optimised parameters of Process S in the regular-wheel (RW) and enforced wakefulness (EW) groups (pooled).* **a-c)** Mean (red line) and individual animal values of the parameters gc, rs, S_U_ in the frontal (Fro) and occipital (Occ) derivations in the RW and EW groups (pooled). ts= 4 s; n=14; red area: 95-% CI, blue area=1 SD; grey circles represent individual animals. The significance of the difference in parameter values between derivations was assessed with a non-parametric Wilcoxon signed-rank test; p-values are indicated above each plot. *Note: Parameters of the EW group, although referred to as ‘EW’, were optimised on the first baseline day of this group (see Figure 5a and Methods).* *RW and EW conditions could be pooled as their baseline conditions were identical. The complex-wheel (CW) results are not referred to here as their parameters were taken from their two baseline days, i.e. the RW condition (see Figure 5a).* SWA= slow-wave activity, NREMS= non-rapid eye movement sleep.

**Supplementary figure S6.** *Long wake bout duration does not affect the ability of the model to predict subsequent slow-wave activity (SWA) levels.* **a)** Correlation between long wake bout durations (day 2 only) and the absolute difference between simulated and empirical SWA levels in non-rapid eye movement sleep (NREMS) epochs in the 40 min following long wake bouts, in the frontal (top) and occipital (bottom) derivations. Regular wheel (RW) & complex wheel (CW): n=6, enforced wakefulness (EW): n=7. **b)** Correlation between long wake bout durations (day 2 only) and the mean SWA levels in NREMS epochs in the 40 min following long wake bouts. RW & CW: n=6, EW: n=7. In a) and b) the lines were fitted with a least-square algorithm. To prevent bias in the correlation, 1 RW individual and 1 CW individual with very short wake bout durations (≤ 1 h) were excluded from this analysis. BL= baseline, sim= simulation.

**Supplementary Figure S7.** *Wake (resp. non-rapid eye movement sleep (NREMS)) EEG undergoes changes across (resp. after) prolonged waking.* **a)** Wake EEG spectra during long wake bouts in the frontal and occipital derivations across the 3 conditions. In each condition, n=7; mean ± SEM. ANOVA revealed a significant main effect of frequency in both derivations (Frontal (Fro) & Occipital (Occ): p<0.001), but a main effect of condition was only significant in the frontal derivation (Fro: p=0.014, Occ: p=0.542). There was a significant interaction in both derivations however (Fro & Occ: p<0.001). In the frontal derivation, post-hoc testing with a Bonferroni correction revealed that the enforced wakefulness (EW) group was significantly different from the complex wheel (CW) group (p=0.019; EW vs RW: p=0.055). Independent t-tests were performed across frequencies; p-values<0.1 are reported below the graphs. **b)** EEG power spectra in the last hour of the long wake bout (day 2 only) expressed as a % of the first hour of the corresponding bout. Regular wheel (RW) and CW: n=6, EW: n=7; mean ± SEM. ANOVA revealed, in both derivations, a significant main effect of frequency (p<0.001), but no main effect of condition (Fro: p=0.334, Occ: p=0.086) and a significant interaction frequency*condition (p<0.001). Post-hoc testing was performed with independent sample t-tests for RW vs. CW on the one hand and RW vs. EW on the other hand. p-values < 0.1 are reported below the figures. ANOVA tests to compare the first and last hours of waking revealed that those hours were significantly different in all 3 conditions and in both derivations, except for the RW frontal (Fro: RW: p=0.199, CW: p=0.005, EW: p=0.092; Occ: RW: p=0.007, CW: p=0.001, EW: p=0.008). **c)** EEG spectra in NREMS during the hour following long wake bouts as a % of the hour preceding wake (day 2 only). RW and EW: n=7, CW: n=6; mean ± SEM. ANOVA tests revealed, in both derivations, a significant main effect of frequency (p<0.001), but no main effect of condition (Fro: p=0.805, Occ: p=0.876) and a significant interaction frequency*condition (p<0.001). Post-hoc testing was performed with independent sample t-tests for RW vs. CW on the one hand and RW vs. EW on the other hand. p-values < 0.1 are reported below figures. ANOVA tests to compare the hours preceding and following long wake bouts revealed that those hours were significantly different in all 3 conditions and in both derivations, except for the EW occipital (Fro: RW: p=0.001, CW: p=0.001, EW: p<0.001; Occ: RW: p=0.003, CW: p=0.001, EW: p=0.154). Note that for clarity and conciseness, no direct comparison between the EW and CW cohorts was performed here, especially as this would not have provided information for the question addressed.

**Supplementary figure S8.** *Correlation between empirical slow-wave activity (SWA) in day 2 after long wake bouts* (mean over the 40 min following long wake bouts (LWB) – expressed as % of previous baseline (BL) day) and the absolute error in the same 40 min; Regular wheel (RW): n=6, Complex wheel (CW): n=6, Enforced wakefulness (EW): n=7. Lines were fitted with a least-square algorithm. To prevent bias in the correlation, 1 RW individual and 1 CW individual with very short wake bout durations (≤ 1 h) were excluded from this analysis. sim= simulation.

**Supplementary figure S9.** *Time spent running on a wheel during long wake bouts does not affect subsequent slow-wave activity (SWA) levels or the accuracy of the simulations.* **a)** Correlation between % of time spent running during long wake bouts (day 2 only) and the absolute difference between simulated and empirical SWA levels in non-rapid eye movement sleep (NREMS) epochs in the 40 min following long wake bouts, in the frontal (top) and occipital (bottom) derivations. Regular wheel (RW) & Complex wheel (CW): n=6, Enforced wakefulness (EW): n=7. **b)** Correlation between % of time spent running in long wake bout (day 2 only) and the mean SWA levels in NREMS epochs in the 40 min following wake bouts. RW & CW: n=6, EW: n=7. In a) and b) the lines were fitted with a least-square algorithm. To prevent bias in the correlation, 1 RW individual and 1 CW individual with very short wake bout durations (≤ 1 h) were excluded from this analysis. BL= baseline, sim=simulation.
